# Sustainable Materials for Energy

**DOI:** 10.3390/nano15181388

**Published:** 2025-09-10

**Authors:** Filippo Agresti, Giuliano Angella, Humaira Arshad, Simona Barison, Davide Barreca, Paola Bassani, Simone Battiston, Carlo Alberto Biffi, Maria Teresa Buscaglia, Giovanna Canu, Francesca Cirisano, Silvia Maria Deambrosis, Angelica Fasan, Stefano Fasolin, Monica Favaro, Michele Ferrari, Stefania Fiameni, Jacopo Fiocchi, Marco Fortunato, Donatella Giuranno, Parnian Govahi, Jacopo Isopi, Francesco Montagner, Cecilia Mortalò, Enrico Miorin, Rada Novakovic, Luca Pezzato, Daniela Treska, Ausonio Tuissi, Barbara Vercelli, Francesca Villa, Francesca Visentin, Valentina Zin, Maria Losurdo

**Affiliations:** 1 CNR-ICMATE National Research Council, Institute of Condensed Matter Chemistry and Technologies for Energy, Corso Stati Uniti 4, 35127 Padova, Italy; filippo.agresti@cnr.it (F.A.); simona.barison@cnr.it (S.B.); davide.barreca@cnr.it (D.B.); simone.battiston@cnr.it (S.B.); silviamaria.deambrosis@cnr.it (S.M.D.); angelicafasan@cnr.it (A.F.); stefano.fasolin@cnr.it (S.F.); monica.favaro@cnr.it (M.F.); stefania.fiameni@cnr.it (S.F.); jacopo.isopi@cnr.it (J.I.); francesco.montagner@cnr.it (F.M.); cecilia.mortalo@cnr.it (C.M.); enrico.miorin@cnr.it (E.M.); luca.pezzato@cnr.it (L.P.); danielatreska@cnr.it (D.T.); francesca.visentin@cnr.it (F.V.); valentina.zin@cnr.it (V.Z.); 2 CNR-ICMATE National Research Council, Institute of Condensed Matter Chemistry and Technologies for Energy, Via R. Cozzi, 53, 20125 Milano, Italy; giuliano.angella@cnr.it (G.A.); humairaarshad@cnr.it (H.A.); parniangovahi@cnr.it (P.G.); barbara.vercelli@cnr.it (B.V.); 3 CNR-ICMATE National Research Council, Institute of Condensed Matter Chemistry and Technologies for Energy, Via Previati 1/e, 23900 Lecco, Italy; paola.bassani@cnr.it (P.B.); carloalberto.biffi@cnr.it (C.A.B.); jacopo.fiocchi@cnr.it (J.F.); ausonio.tuissi@cnr.it (A.T.); francesca.villa@cnr.it (F.V.); 4 CNR-ICMATE National Research Council, Institute of Condensed Matter Chemistry and Technologies for Energy, Via De Marini 6, 16149 Genova, Italy; mariateresa.buscaglia@cnr.it (M.T.B.); giovanna.canu@cnr.it (G.C.); francesca.cirisano@cnr.it (F.C.); michele.ferrari@cnr.it (M.F.); marco.fortunato@cnr.it (M.F.); donatella.giuranno@cnr.it (D.G.); rada.novakovic@cnr.it (R.N.)

**Keywords:** phase-change materials, carbon dots, gCN, TiAlN, ferroelectric ceramics, iron castings, Cu-based alloys, Ni-based alloys, amphiphobic coatings, hydrogen permeation barriers, hydrogen membranes, thermal energy storage, heat exchangers, HiPiMS, additive manufacturing, LCA

## Abstract

The sustainable production of energy without environmental footprints is a challenge of paramount importance to satisfy the ever-increasing global demand and to promote economic and social growth through a greener perspective. Such awareness has significantly stimulated worldwide efforts aimed at exploring various energy paths and sources, in compliance with the ever more stringent environmental regulations. Research advancements in these fields are directly dependent on the design, fabrication, and implementation of tailored multi-materials for efficient energy production and harvesting and storage devices. Herein, we aim at providing a survey on the ongoing research activities related to various aspects of functional materials for energy production, conversion, and storage. In particular, we present the opportunities and the main open challenges related to multifunctional materials spanning from carbon-based nanostructures for chemical energy conversion, ferroelectric ceramics for energy harvesting, and phase change materials for thermal energy storage to metallic materials for hydrogen technologies, heat exchangers for wind energy, and amphiphobic coatings for the protection of solar panels. The relevance of designing tailored materials for power generation is also presented. Finally, the importance of applying life cycle assessment to materials is emphasized through the case study of AlTiN thin films.

## 1. Introduction

In an era defined by escalating energy demands and the urgent need to address climate change, the pursuit of sustainable solutions stands at the forefront of scientific and technological innovation. Central to this endeavor is the development and deployment of sustainable materials with minimal environmental impact to revolutionize the way we generate, store, and consume energy. Energy material research encompasses diverse materials and technologies critical for addressing global energy challenges. Their applications span from solar panels and wind turbines to fuel cells, batteries, and energy-efficient building materials.

Solar panels are moving silicon, III-V and II-VI compounds, and quantum dots [1], as well as organic photovoltaics utilizing conjugated polymers or small molecules as active layers and innovative hybrid organic–inorganic perovskite compounds, to the fourth-generation solar cells [2] to efficiently convert sunlight into electricity.

Wind turbines are constructed from recyclable steel, new alloys, composites, and even eco-friendly resins to harness wind power [3].

Energy storage is also pivotal in achieving the EU’s climate neutrality targets. Notably, advances in nanomaterials and bidimensional (2D) materials have introduced sustainable options for energy harvesting and storage. There has been a growing focus on employing eco-sustainable biomass-derived biopolymers and native materials as a viable alternative to traditional energy storage applications. Biopolymer-based energy devices, like batteries, supercapacitors, electrode materials, and ion-exchange membranes, a novel and eco-conscious approach, hold great potential for flexible and smart electrochemical energy storage and conversion devices, owing to their affordability, environmental sustainability, and biodegradability [4]. The recent development in the field of 2D materials, including both graphene and other layered systems, such as phosphorene, has shown promise for supercapacitors and batteries [5,6]. Graphene-based supercapacitors offer rapid charging and discharging cycles, significantly enhancing the performance and sustainability of energy storage systems. MXenes are also a relatively new class of two-dimensional materials, typically composed of transition metal carbides, nitrides, or carbonitrides, with exceptional electrical conductivity, mechanical flexibility, and rich surface chemistry that make MXenes highly promising for various energy-related applications, such as supercapacitors, batteries, and thermoelectric generators [7,8].

Phase-change materials (PCMs) made from bio-based fatty acids or salts are increasingly used in energy-efficient buildings for thermal energy storage [9]. By absorbing and releasing latent heat during phase transitions, these materials help maintain comfortable indoor temperatures and reduce the reliance on fossil-fuel-based heating and cooling.

Research into lead-free piezoelectric ceramic materials can help convert mechanical stress into electrical voltage, enabling the harvesting of vibrational energy from ambient sources [10].

Simultaneously, the exploitation of alternative resources has emphasized the strategic importance of molecular hydrogen (H_2_) as an energy vector to tackle the global energy crisis. A promising method for producing green hydrogen [11], as opposed to gray hydrogen [12] derived from fossil fuels, is the electrolysis of water driven by natural sunlight. However, the overall process efficiency remains suboptimal due to the sluggish oxygen evolution reaction (OER), necessitating the development of green and economically viable catalysts as alternatives to the current noble metal-based ones, thereby motivating research into novel catalytic materials.

The development and modulation of these materials through appropriate fabrication and processing methods are indeed the Holy Grail for meeting current technological requirements and ensuring significant advancements from laboratory to industrial applications in the context of improved sustainability. This scenario could be achieved in the near future, provided that the developed materials and fabrication methods meet the criteria of eco-friendliness, reproducibility, integration with established industrial processes, and economic sustainability.

**Figure 1:**
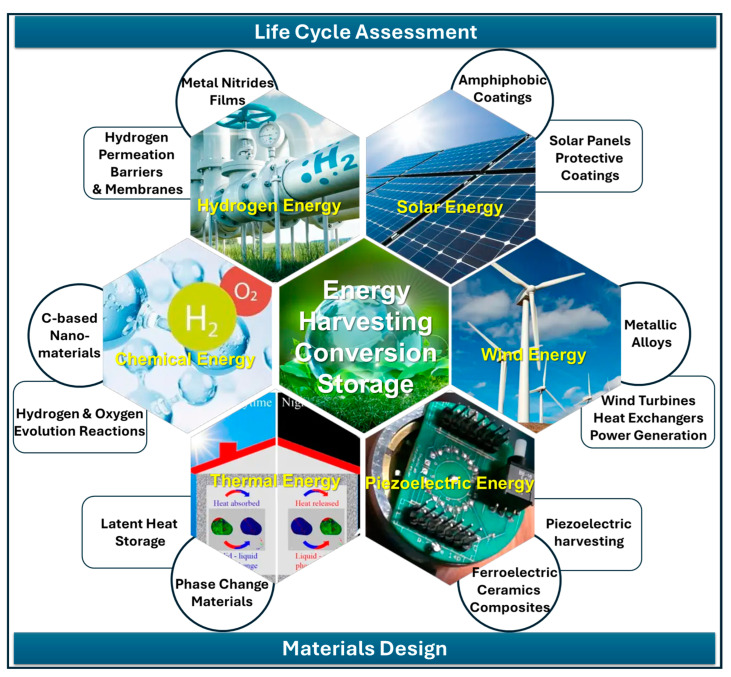
Schematic synoptic overview of materials for energy targeted in this work.

This work aims to enlarge the current landscape of sustainable materials for energy, discussing ongoing activities related to the development of advanced materials and technologies for sustainable energy production, encompassing bulk systems, thin films, and nanostructures, as well as strategies for efficient energy production, conversion, and storage. By examining both recent breakthroughs and persistent critical challenges that hamper widespread adoption, such as scalability, cost, and integration with existing infrastructure, this study aims to illuminate the path toward a resilient and environmentally conscious energy future. The related issues are presented and discussed in different sections through the critical analysis of selected case studies summarized in Figure 1.

Specifically, Section 2 discusses carbon-based nanostructures for thermochemical energy conversion and water splitting, with a focus on biomass-derived carbon dots and graphitic carbon nitride, a promising material for solar-assisted hydrogen production. Section 3 is dedicated to thermal energy storage using phase-change materials, which are crucial for improving the performance and energy efficiency of buildings. Section 4 focuses on research outcomes related to hydrogen barrier coatings and permeation membranes based on metallic nitride thin films, which are important for hydrogen transportation and storage. Ceramics materials and composites have also emerged as attractive candidates due to their mechanical, thermal, and chemical stability, as well as their piezoelectric properties. Relevant examples of energy harvesting from ambient sources and energy storage using ferroelectrics are contained in Section 5. Section 6 focuses on metallic materials, particularly alloys for specific applications in advanced heat exchangers and wind energy. Section 7 is devoted to amphiphobic and self-cleaning coatings for the protection of solar panels. The selection and engineering of these materials are guided by principles such as life cycle assessment, resource conservation, and the reduction in toxic byproducts, all of which are integral to achieving a circular economy within the energy sector. Therefore, Section 8 presents the relevance of applying life cycle assessment (LCA) to the development of materials for energy, discussing the case study of AlTiN thin films used in hydrogen technologies. Some considerations of recycle and reuse are discussed in Section 9. Finally, an outlook and development prospects are presented, aiming to provide pointers for innovative research efforts in this field.

## 2. Carbon-Based Nanomaterials for Chemical Energy Conversion

Energy conversion is a critical aspect of harnessing renewable energy, enabling its transformation into chemical energy and storage in the form of hydrogen produced by the electrochemical splitting of water. This stored energy can be utilized in devices such as fuel cells when needed. Recent advances have highlighted the importance of developing green electrocatalysts for both the hydrogen evolution reaction (HER) and the oxygen evolution reaction (OER) in water splitting. In this context, carbon-based nanomaterials are very attractive frontrunners due to their electrical, mechanical, and optical properties, easily tunable morphologies, high surface area, rich surface chemistry, multiple porosity, high thermal and chemical stabilities, and intrinsic low toxicity as well as for the green character and economic viability. While several papers have reported on the use of graphene, carbon nanotubes, and fullerenes for both energy storage and conversion [13], this section will focus on carbon dots from biomasses and graphitic carbon nitride-based systems as it is highly desirable to have the production of carbon-based materials from abundant, eco-friendly, scalable, and low-cost sources.

### 2.1. Biomass-Derived Carbon Dots for Solar Energy, Thermal Conversion, and Hydrogen Evolution Reaction

Carbon-based dots (CDs) are a class of fluorescent nanomaterials consisting of a carbon core with sizes below 10 nm enveloped by different surface functionalities. Their use has recently rapid expanded in energy production [14,15,16,17], particularly for hydrogen generation [18] and solar–thermal conversion [15], playing a crucial role in the design and development of organic solar cells (OSCs) and dye-sensitized solar cells (DSSCs) [16,19,20].

**Figure 2:**
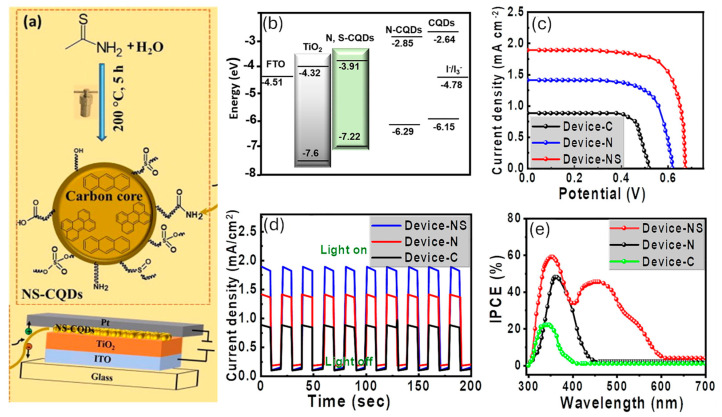
(**a**) Scheme of N, S-CQDs formation and solar cell assembly. (**b**) Energy level diagram, (**c**) J-V curves, (**d**) transient photocurrent density, and (**e**) IPCE spectra of all devices. Reproduced with permission from ref. [17]. Copyright 2022 Elsevier Ltd.

This broad perspective of utilization is empowered by their favorable properties including high photostability, good water solubility, and intrinsic low toxicity [21,22,23,24]. Furthermore, the possibility of doping their carbon cores with heteroatoms (oxygen, nitrogen, sulfur, phosphorus, boron, etc.) combined with the presence of functional groups on their surface provides a plethora of active sites contributing to the improvement in their electro-and photocatalytic performances. As a matter of fact, by suitable CD design and engineering, it is possible to modulate their electronic structure, band gap, and photoluminescence properties, paving the way to the obtainment of functional devices with tailored performances. A representative example is offered by the use of N and S-co-doped CDs (N, S-CDs) as a sustainable sensitizer/photoactive layer in quantum dot-sensitized TiO_2_-based solar cells (TQDSCs) (Figure 2) [17]. Compared to the devices sensitized with undoped-CDs and N-CDs, those sensitized with N,S-CDs showed improved performances, reaching a short circuit current density (J_sc_) of 1.89 mA/cm^2^ and a PCE of 0.92%, against the J_sc_ of 0.88 mA/cm^2^ and 1.41 mA/cm^2^ and PCE of 0.28% and 0.57%, of the undoped and N-doped CDs, respectively. These results were ascribed to the proper band alignment of N, S-CDs (Figure 2), resulting in an electron transfer to the wide band gap semiconductor.

**Figure 3:**
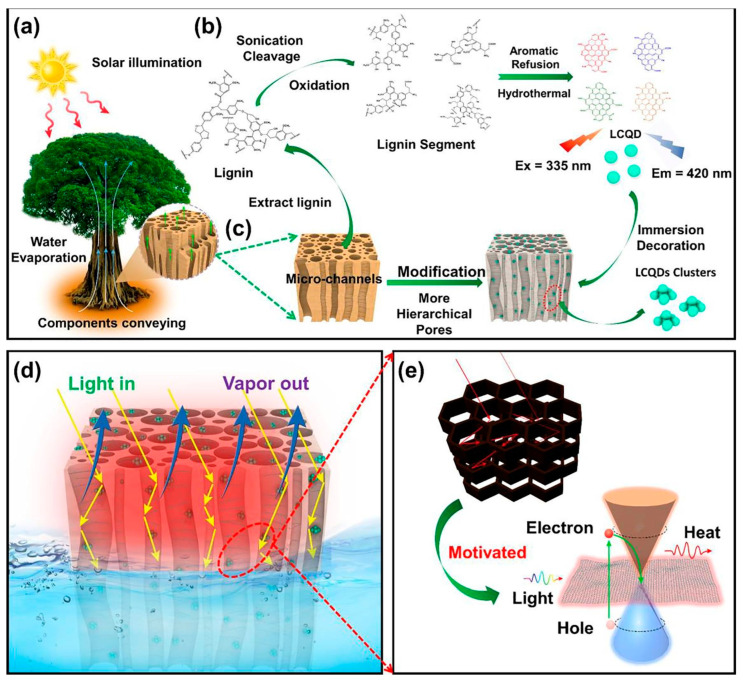
(**a**) Schematic of a grown tree and solar-motivated transpiration. (**b**) Delignification of wood and hydrothermal fabrication of LCDs. (**c**) Decoration of LCDs onto the channels of DW. (**d**) Schematic for LCDs-DW showing the photothermal evaporation system function under natural sunlight. (**e**) Graphical illustration of the carbon-based hexatomic ring structure that can enhance light absorption and photothermal conversion in LCDs. Reprinted with permission from ref. [27]. Copyright 2021 Elsevier Ltd.

Furthermore, the integration of CDs into materials used in solar–thermal conversion improves the energy generation process [25,26]. Ho et al. [27] developed a new sustainable solar-driven photothermal evaporation (PE) system based on the processes of evaporation and condensation for water purification (Figure 3). The PE system is entirely sustainable as it consists of delignified wood (DW) with incorporated CDs obtained from lignin (LCDs). DW and LCDs are, respectively, used for vapor transportation and photothermal conversion. The best evaporation efficiency was 79.5%, demonstrating the superior application potential of the system. By contrast, the evaporation efficiencies of water, wood, and DW reached only 16.2%, 54.2%, and 47.4%, respectively, indicating their insufficient thermal conduction and low photothermal conversion. The authors attributed the improved photothermal performance to the presence of CDs, which increase light absorption.

In addition, CDs are also employed as support for catalysts in HER and OER for water splitting [18,28,29]. Fan et al. [30] reported the use of Pt supported on CDs for HER. The authors incorporated the Pt-CDs system onto CdS nanorods to optimize a Pt-CDs@CdS ternary photocatalyst, which exhibited a photocatalytic hydrogen evolution (PHE) activity of about 46.10 mmol·h^−1^g^−1^, that was about 68.8, 14.5, and 2.9 times higher than that of pure CdS nanorods, CDs@CdS, and Pt@CdS, respectively.

Moreover, it exhibited excellent stability in photocatalytic water splitting, maintaining a PHE rate of more than 38 mmol·h^−1^g^−1^ even after 60 h of continuous irradiation. The authors ascribed the enhanced catalytic performance to the synergistic effect of the combination of Pt on CDs. Conversely, Tian et al. [30] demonstrated that incorporating CDs into the FeNi_3_ catalyst can finely tailor its morphology and conductivity, determining a and strong synergistic coupling effect. The optimized catalyst shows extraordinary electrocatalytic performance towards OER by delivering a current density of 10 mA cm^−2^ with the overpotential of 238 mV, as well as a small Tafel slope of 48.7 mV dec^−1^.

**Figure 4:**
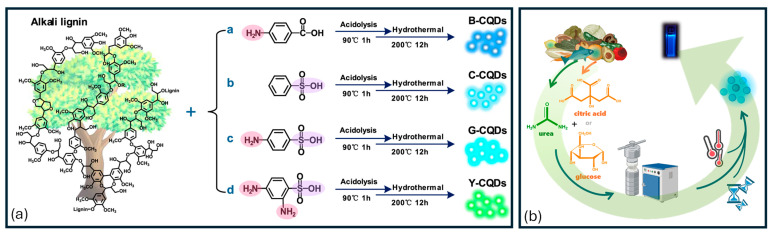
(**a**) Illustration of the formation process of CDs from alkali lignin treated with acid additives by hydrothermal treatment. Reproduced with permission from ref. [40]. Copyright 2021 American Chemical Society. (**b**) Illustration of CDs’ synthesis from small molecules extracted from agro-food waste. Reprinted with permission from ref. [36]. Copyright 2024 Wiley-VCH Verlag GmbH & Co. KGaA, Weinheim.

For all these applications, the preparation of cost-effective CDs represents a relevant aspect of their sustainability that can be achieved through the development of synthetic approaches spanning from hydrothermal/solvothermal to electrochemical, chemical, and microwave-assisted methods utilizing precursors derived from the recovery of biological and agro-industrial wastes. Recently, a considerable number of research efforts have been devoted to the development of sustainable synthesis approaches based on environmentally friendly precursors derived from biomass and biowaste, as well as the development of novel processes that are both efficient and do not require high levels of external energy [31]. Among the various possible synthetic approaches, the hydrothermal/solvothermal method is particularly suitable for sustainable applications and scale-up for large-scale production [32,33,34,35,36,37]. This approach is simple and cost-effective, and enables the modulation of CD particle size, functional groups, and optical properties [38], as well as significative production yields [39]. Zhu et al. [40] employed alkali lignin and acid additives to develop a two-step sustainable hydrothermal approach to prepare tunable fluorescent CDs (Figure 4a).

The obtained CDs exhibit an intense color evolution from blue to yellowish green depending on the employed acid additives. This CDs’ fluorescence emission has been proven to increase the conversion efficiency of C-dot-sensitized solar cells. In a recent study [36], we employed small molecules, like citric acid, glucose, urea, and others, extracted from agro-food wastes for the hydrothermal synthesis of CDs (Figure 4b). We reported the influence of the process parameters on the properties of the obtained CDs to develop a sustainable strategy that could be reliable and reproducible, showing that the process temperature seems to be the “key parameter” that influences the properties of CDs. These synthetic approaches are still limited to the laboratory scale and their scale-up for sustainable industrial applications is still an ongoing research area. Consequently, the commercialization of CDs is still limited to few companies that offer small amounts of customized CDs for laboratory applications in their catalog. Furthermore, the full comprehension of the synthesis mechanism, including the process conditions and the influential parameters, and the development of adequate purification strategies are other important issues to consider for the preparation of high-quality CDs with suitable properties for energy applications. Despite these challenges, CDs represent a great promise in the advancement of clean energy technologies by sustainably addressing global energy needs.

### 2.2. Nanoarchitectures Based on gCN for Sustainable Energy Production

**Figure 5:**
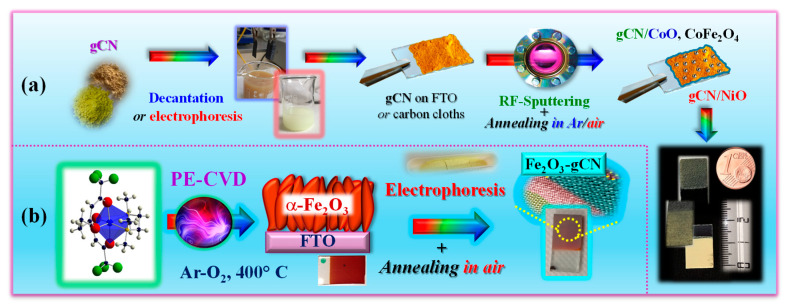
(**a**) Sketch of the preparation route of graphitic carbon nitride systems containing ultra-dispersed CoO, CoFe_2_O_4_, and NiO. For Co-containing oxides, decantation of gCN on fluorine-doped tin oxide (FTO) glasses was followed by radio frequency (RF) sputtering under mild conditions for CoO or CoFe_2_O_4_ functionalization, followed by annealing in Ar. In the case of NiO, the target materials were obtained by mixing gCN and Ni(II) acetate powders, subsequent electrophoresis on carbon cloths, and annealing in air. (**b**) Strategy to fabricate Fe_2_O_3_ specimens functionalized with gCN: plasma-enhanced chemical vapor deposition (PE-CVD) of iron(III) oxide followed by gCN introduction via electrophoresis and final annealing in air. Reproduced with permission from refs. [48] (Copyright 2023, Elsevier) and [51] (Copyright 2023, The Royal Society of Chemistry).

In the ever-increasing search for economically viable, environmentally friendly, and Vis-light-active photoelectrocatalysts for sustainable energy generation, graphitic carbon nitride (gCN), a 2D metal-free *n*-type semiconductor, has attracted an enormous interest [41,42,43]. The latter is motivated by various gCN attractive properties, encompassing its band gap (*E*_G_ ≈ 2.7 eV) and Vis light harvesting properties, chemical/thermal stability, non-toxicity, low cost, and tunable composition/electronic structure [44,45]. In spite of these advantages, bare gCN suffers from a limited active site amount, a low surface area, and a relatively fast recombination of photogenerated charge carriers [46,47]. Among the possible solutions to alleviate such shortcomings, the combination of gCN with suitable metal oxides in tailored heterostructures, allowing also to benefit from an improved light absorption, is as an extremely attractive toolkit. In this regard, gCN can be utilized either as the main system component (Figure 5a) [48,49,50] or as a functionalizing agent grafted onto suitable oxide partners (Figure 5b) [51,52]. A judicious selection of system components and supporting scaffolds, along with the implementation of versatile routes to nanosystems, paves the way for potential material integration into functional devices for real-world applications [53].

Herein, we focus on the most relevant characteristics of representative systems belonging to categories (a) and (b), along with their performances in the oxygen evolution reaction (OER), the bottleneck of water splitting for yielding green hydrogen [48,49,50,51].

**Figure 6:**
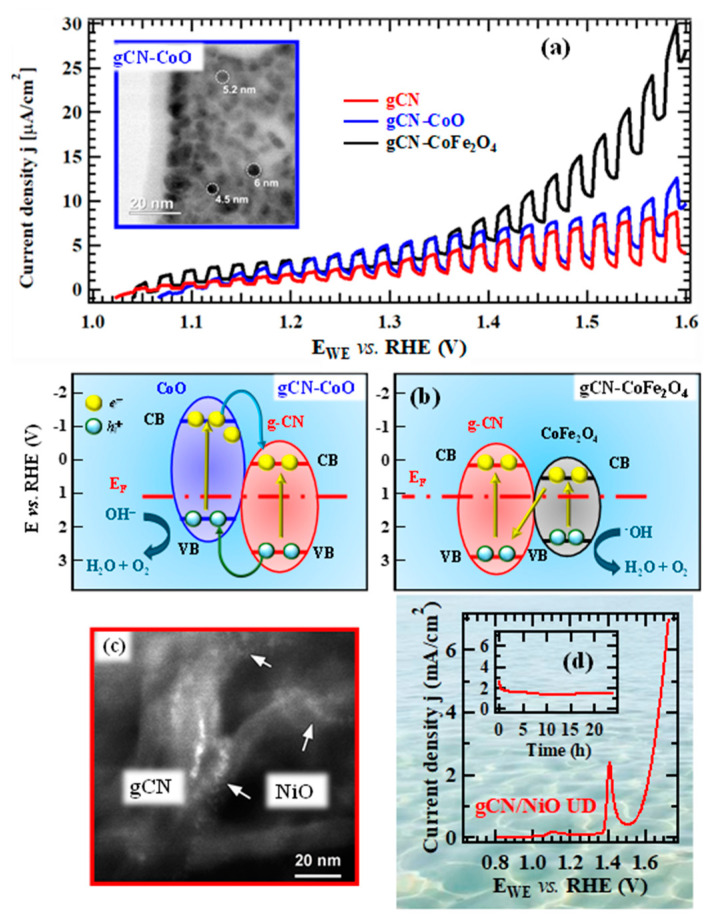
(**a**) Photoactivated OER performances in aqueous KOH (0.1 M, pH = 12.9) of bare and functionalized gCN samples. RHE = reversible hydrogen electrode. (**a**) Chopped light linear sweep voltammetries (LSVs). Inset: representative transmission electron microscopy (TEM) image for gCN-CoO specimen. (**b**) Sketch of interfacial band energetics for gCN-CoO (**left**) and gCN-CoFe_2_O_4_ (**right**). E_F_ = Fermi-level energy; CB and VB = conduction and valence band edges, respectively [48]. (**c**) High-resolution TEM micrograph of gCN decorated with ultra-dispersed NiO, indicated by white arrows. (**d**) LSV curve under irradiation for gCN/NiO nanocomposite in Adriatic seawater (pH = 13.58). Inset: CA scan recorded at 1.6 V vs. RHE [50]. Reproduced with permission from refs. [48] (Copyright 2023, Elsevier) and [50] (Copyright 2024, Wiley-VCH).

In the course of our studies on gCN-based electrocatalysts, we have initially focused our attention on the functionalization of supported gCN with a minimum amount of complementary Co-containing catalysts capable of enhancing the ultimate OER photoactivity (Figure 5a). A multi-technique investigation through the use of forefront analytical tools [48] ascertained the formation of pure materials featuring highly dispersed oxide nanoparticles, with an intimate contact between the system components. This is exemplified by the micrograph displayed in the inset of Figure 6a, that shows evenly distributed and randomly oriented spherical nanoparticles [mean diameter = (5 ± 1) nm] comprising cubic CoO as the sole crystalline phase. Additional analyses evidenced the formation of open area materials, characterized by a non-negligible content of uncondensed amino groups (-NH_x_, with x = 1, 2), increasing according to the sequence gCN < CN-CoO < gCN-CoFe_2_O_4_. The parallel increase in defects resulting from -NH_x_ presence, which can act as capturing sites suppressing detrimental electron–hole recombination, is a favorable issue to promote an improved system photoactivity [54,55]. In fact, chopped light linesr sweep voltammetry (LSV) analyses (Figure 6a) displayed photocurrents progressively increasing upon going from bare gCN to gCN-CoFe_2_O_4_ that turned out to be the best performing photoanode. In parallel, a progressive increase in material long-term stability was evidenced by chronoamperometric (CA) analyses. This attractive performance improvement can be attributed to the concurrence of different phenomena, including the increased nitrogen defect content (see above), enhanced radiation absorption, and effective formation of junctions boosting electron–hole separation. Regarding g-CN-CoO, the formation of *p*-*n* junctions must be considered (Figure 6b, left panel), since electrons must reach the external circuit through g-CN flakes, in contact with the FTO substrate. In a different way, in the case of gCN-CoFe_2_O_4_, a Z-scheme junction (Figure 6b, right panel) is the only possibility accounting for the observed system behavior. Photopotential measurements evidenced an improved electron–hole separation in the latter case, thus explaining the corresponding higher photoactivity [48].

In an alternative approach, a straightforward preparation route to gCN OER photoelectrocatalysts supported on flexible carbon cloths, integrating highly dispersed NiO as co-catalyst, involved a single-step electrophoretic process (see Figure 5a), followed by a final thermal treatment in air. The use of such substrates instead of conventional FTO is appealing thanks to their stability, conductivity, large area, and favorable interactions with gCN, allowing to eliminate binders or additives that may compromise the ultimate performances [50,56]. A suitable control of operating conditions enabled to obtain ultra-dispersed NiO in the hosting gCN, with a high density of *p*-*n* NiO/gCN heterojunctions (similarly to the case of Figure 6b, left panel). In fact, TEM images (Figure 6c) revealed that gCN flake edges were decorated by tiny bright dots, thus highlighting a “quasi-atomic” NiO dispersion, an attractive issue in view of the target application [50]. In fact, the obtained gCN/NiO specimens are demonstrated as noble metal-free, cost-effective, and efficient OER photoelectrocatalysts not only in alkaline freshwater, but even in seawater. The pertaining OER functional tests (Figure 6d), never reported up to date for homologous materials, highlighted that, despite the corrosive reaction environment, the system delivered an appreciable photocurrent and featured a very attractive stability, as also demonstrated by *post-operando* chemicophysical analyses [50]. Overall, these outcomes open intriguing perspectives for sustainable energy production from abundant and low-cost solar light and seawater. Such a prospected utilization, of strategic interest for an eventual decarbonization of the current energy portfolio, is also reinforced by the possibility of manufacturing anodes selective towards OER against the competitive hypochlorite formation, kinetically favored due to the high salt concentration in seawater [57].

A relevant example in this regard is Fe_2_O_3_/graphitic carbon nitride (gCN) seawater oxidation photoelectrocatalysts obtained via PE-CVD of hematite, the most thermodynamically stable Fe_2_O_3_ polymorph, followed by gCN anchoring through a fast electrophoretic deposition, and final annealing in air.

**Figure 7:**
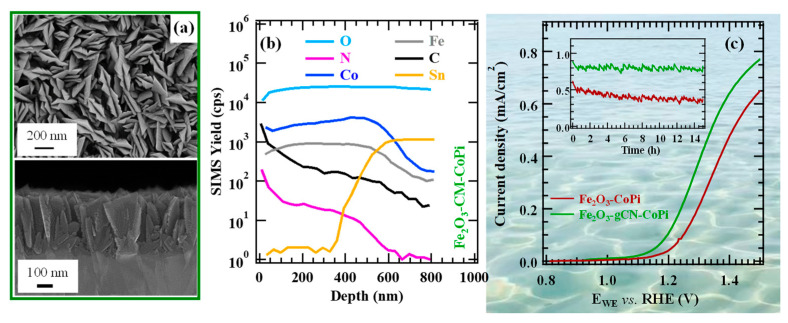
(**a**) Plane-view (top) and cross-sectional (bottom) scanning electron microscopy (SEM) images for Fe_2_O_3_-gCN system. (**b**) Secondary ion mass spectrometry (SIMS) depth profile for Fe_2_O_3_-gCN sample after electrodeposition of Co(II) phosphate (CoPi). (**c**) LSV curve under irradiation for gCN/NiO specimen in Adriatic seawater (pH = 13.58). Inset: CA scan at 1.45 V vs. RHE. Reproduced with permission from ref. [51] (Copyright 2023, The Royal Society of Chemistry).

The resulting materials possessed a porous lamellar morphology with a high active area that did not undergo any relevant modification after functionalization with gCN (Figure 7a) or after introduction of CoPi, a well-known oxidation co-catalyst [49,50]. In-depth analyses (Figure 7b) underscored a gCN dispersion throughout the underlying iron(III) oxide, thanks to the open morphology of the latter, and a close resemblance of iron and cobalt profiles after the introduction of CoPi, whose even distribution synergistically contributed to an additional performance improvement. LSV curves (Figure 7c) highlighted a net photocurrent improvement after the introduction of carbon nitride that was traced back to the formation of type-II Fe_2_O_3_/gCN junctions (the accumulation of electrons and holes in Fe_2_O_3_ CB and gCN VB, respectively). The introduction of CoPi and its OER electrocatalytic activity yielded a favorable activity improvement, with no competitive hypochlorite formation. The actual performances are in line, or even better, than those exhibited in seawater splitting by other Fe_2_O_3_- or gCN-containing materials [51].

These results, along with the system long-term stability, improved by gCN introduction (Figure 7c, inset), play an important role towards the exploitation of the natural capital for clean energy production, in full compliance with UN Agenda 2030 goals for sustainable development.

## 3. Phase-Change Materials as Innovative Approach to Thermal Energy Storage

**Figure 8:**
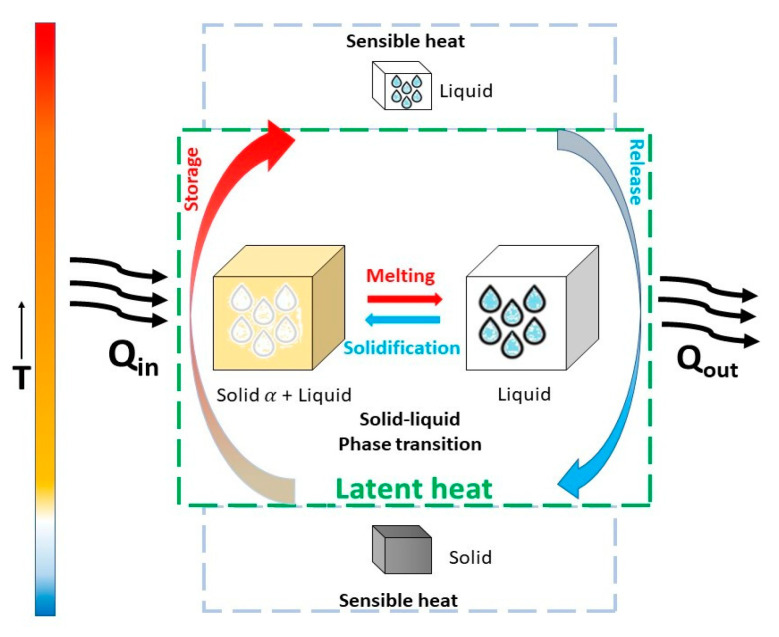
Schematic representation of heat storage through solid–liquid phase transition.

Thermal energy storage (TES) systems stock thermal energy by heating or cooling a storage medium. TES technologies include sensible heat storage, latent heat storage, and thermochemical energy storage. These methods store energy as heat in various mediums such as water, molten salt, or phase-change materials (PCMs). Among these, latent heat storage (LHS) involves PCMs that absorb or release energy during phase changes, such as solid-to-liquid, liquid-to-gas, or solid-to-solid transitions, as schematized in Figure 8. The more energetically favorable phase changes are solid/liquid transformations (Figure 8) that exploit the latent heat released upon melting and absorbed during the solidification of materials, eventually complemented by sensible heat at lower or higher temperatures. This method allows for high-density energy storage in a compact space, which makes PCMs particularly valuable in various energy applications, from renewable energy integration to building efficiency and beyond. Herein, we focus on the potential of two classes of PCMs, namely novel metallic PCMs and PCM emulsions.

### 3.1. New Frontiers by Moderate-to-High-Temperature Metallic Phase-Change Materials

LHS PCMs can be sorted into different categories according to the three main criteria of chemical nature, phase change state, and temperature range of phase transition, $T_{m}$ (Table 1). Focusing on the phase transition temperature, a low temperature range for $T_{m}$ < 100 °C is of interest for building applications, whereas moderate-to-high temperatures, i.e., 100 °C < $T_{m}$ < 1000 °C, are relevant for energy and industrial applications.

Jankowski et al. [59] have reviewed, correlated, and presented graphically the phase-change temperature distribution of more than 700 PCMs in low, medium, and high temperature ranges for the thermal buffering of vehicles. Based on the comparison of thermophysical properties including specific mass and volumetric latent heats, they have identified potential materials for each vehicle system and recommended solid–solid and metallic PCMs for high-energy-density applications.

The optimal properties for different types of PCMs include not only high latent heat and thermal stability, but also low corrosion, non-toxicity, no supercooling, non-flammability, and low cost [60]. From this perspective, at low temperatures, some organic PCMs like paraffins, non-paraffins, and sugar alcohol have been proposed as renewable alternatives although their chemical and thermal stability upon thermal cycling is still challenging. Furthermore, inorganic PCMs (hydrated salts) are non-flammable, abundant, and have high melting point and low volume change. But they are limited to medium-to-long-term thermal management, for example, for electronic applications at a low frequency because of moderate heat transfer rate into phase-change materials.

For medium-to-high temperatures, molten salts including alkali metal nitrates, nitrites, and their mixtures are among the most used PCMs [61]. Despite this, they are affected by their intrinsic low thermal conductivity in the solid phase, yielding low charging/discharging rates.

In this context, metallic PCMs (m-PCMs) are of interest for the medium-to-high temperature range thanks to the high thermal conductivity that can provide fast thermal response. m-PCM are considered as a favorable choice due to their thermal conductivity at least two orders of magnitude higher than that of molten salts [62,63,64], improving latent heat storage capacity by volume and small volume change during phase transition [65,66,67,68].

The primary challenge impeding the utilization of metal-based phase-change materials (m-PCMs) in thermal energy storage (TES) applications is the necessity for adequate confinement during their liquid state. Liquid metals exhibit high corrosivity towards numerous materials. Additionally, the solid–liquid phase transitions are accompanied by volumetric changes, which can induce significant mechanical stresses on the containment media during thermal cycling. To harness the potential of m-PCMs, recent research has concentrated on developing an optimal combination of m-PCMs and their respective containers. Various fabrication methodologies are currently being explored to produce efficient and user-friendly m-PCMs. A comprehensive list of proposed production strategies is presented Table 2.

**Figure 9:**
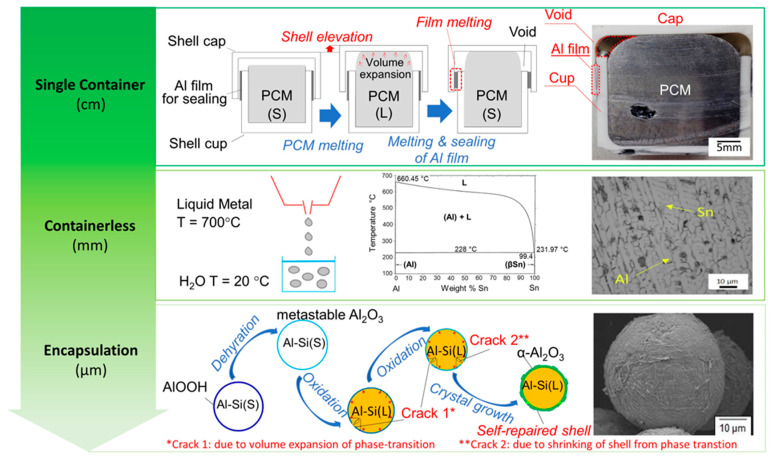
Schematic representation of fabrication approaches.

Three main strategies are pursued, as represented in Figure 9. The most straightforward approach involves designing a single container to house the entire quantity of metallic phase-change materials (m-PCMs). The m-PCMs can be fabricated using conventional metallic material production methods, such as casting, and subsequently placed within the container [68,69,70,71,72,73,74,75,76]. However, several challenges have been encountered with this approach, including thermal stresses, mechanical stability, and corrosion, which arise from chemical reactions between the active PCM and the container material.

The predominant method for producing m-PCMs involves encapsulating small quantities of PCM within capsules, thereby ensuring the effective containment of the liquid phase. Encapsulation is advantageous for latent heat storage due to its increased heat transfer area and protection against environmental fluctuations. Various encapsulation techniques exist, with macro-encapsulation (capsule size > 1 mm) and micro-encapsulation (capsule size between 1 µm and 1 mm) being commonly employed. Micro-encapsulation, compared to macro-encapsulation, offers a faster charging/discharging rate due to the reduced heat transfer distance [89]. However, the smaller mass ratio between PCM and coating results in decreased energy storage density [90].

Encapsulating metallic PCMs present challenges due to the high chemical corrosion of liquid metals with most potential shell materials and volume expansion during phase transition from solid to liquid. The former issue necessitates the use of inert or ceramic-based coatings, while the latter is more significant for PCMs that transition at high temperatures, especially if encapsulated in their solid form at temperatures significantly lower than the operational range. Consequently, container selection must consider sufficient tensile strength and toughness to withstand thermal stresses induced by PCM volume expansion. Most encapsulation processes involve multistep procedures, starting with PCM preparation followed by deposition on the material forming the capsule. These processes can be costly in terms of preparation time and production expenses, limiting the widespread adoption of encapsulated PCMs [78,79,80,82].

**Figure 10:**
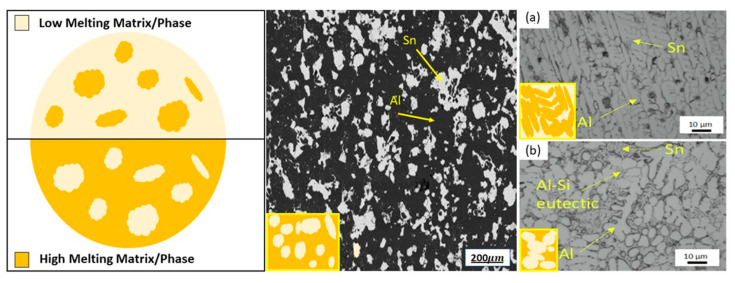
Schematic representation of normal (**upper**) and inverse (**lower**) microstructures (**left**), SEM image of inverted Al-Sn microstructure (**middle**) [83], and optical images of (**a**) Al-Sn and (**b**) Al-Si-Sn (**right**) [84].

The limits of the previous two approaches foster the research for containerless methods that take advantage of the formation of different phases in suitable metallic alloys, where one phase melts at a considerably lower temperature than the others. The latter is the active (PCM) phase. The first studies were performed by Sugo et al. [83] on miscibility gap Al-Sn alloys, in which Sn is the active PCM. The obtained material inherited the mechanical strength and conductivity of metals. The heat storage capabilities of such systems depend on the combination of latent heat (LH) and sensible heat (SH) of the involved phases and can be modulated accordingly. A proper microstructure, the so-called “inverse microstructure” in which the active phase is surrounded by the other phases composed of the metallic PCM, must be obtained (Figure 10). Two directions are being followed to exploit this latter method. One approach is exploring different production methods aiming at an optimal inverse microstructure: initially, these materials were produced by simple powder mixing and compaction, as performed by Sugo et al. [83]. Subsequently, mechanical pre-treatment of powder and ball milling were also experimented, with the aim of improving the mechanical characteristics of the produced PCM [86,87].

More recently, casting processes were proposed, for the production of Al-Sn-based alloys with proper microstructures. Also, advanced 3D printing proved to be quite an effective alternative to produce form-stable m-PCMs with isolated active-phase particles [86], opening a new frontier on tailored shaping of m-PCMs.

We propose that the use of a commercial Al alloy as a substitute of pure aluminum increased the number of microstructural constituents in the system, with a more complex arrangement. Promising results in terms of thermal stability of the produced material were obtained for the investigated Al-Sn based alloys, as confirmed by repeated differential scanning calorimetry (DSC) analyses across the melting temperature range of the low-melting phase. Moreover, the evolution of the microstructural features and phase distributions with thermal cycling was monitored through scanning electron microscopy (SEM). Finally, advanced 3D printing also proved to be quite an effective alternative to produce form-stable m-PCMs with isolated active-phase particles, opening a new frontier on tailored shaping of m-PCMs. Thus, the multitude of processes presented clearly highlight the intense research on m-PCM, on a multitude of materials, sizes, shapes, and temperatures, that can fulfill the requirements of a variety of specific applications.

Research is ongoing on the widening of the types of Al alloys considered and exploring the processability window for such materials by numerical analysis and experimental evaluation.

### 3.2. Phase-Change Material Emulsions for Thermal Energy Storage and Phase Change Materials in Buildings

**Figure 11:**
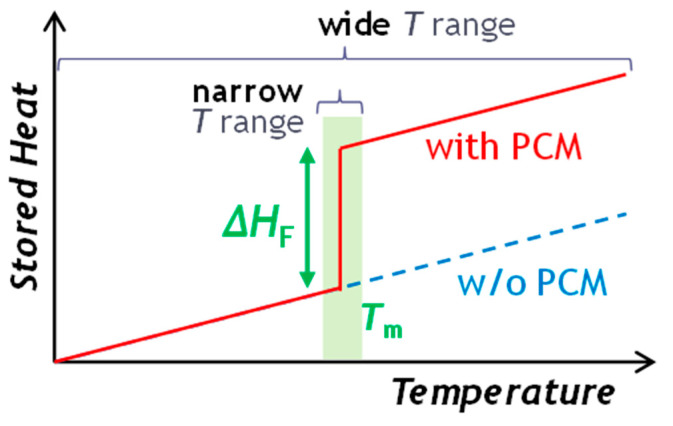
Scheme representing heat storage capacity of PCME.

Heating and cooling in European buildings and industry account for half of the EU’s energy consumption [91]. The cooling demand in Europe is estimated to be over 1.200 TWh cold/year for the next few years. In cooling applications, liquid cooling has been considered the most compelling because of its excellent cooling performance, good long-term reliability, and good shape adaptability [92]. Liquid cooling is a constantly a growing market. As an example, Europe data center liquid cooling market size surpassed USD 650 million in 2021 and is estimated to witness 20% CAGR from 2022 to 2028. Two widely used cooling fluids are water and glycol. Since these refrigerants can only utilize sensible heat to transfer thermal energy, they exhibit a low energy storage density. Therefore, research has been pushed towards more complex systems based on PCM emulsions in refrigerants for the development of high-efficiency heat transfer fluids [93,94]. Phase-change material emulsions (PCMEs) consist of a base fluid, a heat transfer fluid, and an emulsified PCM surrounded by a stabilizing agent, such as surfactants or emulsifiers, immiscible with the base fluid. These emulsions are often engineered to ensure thermal stability, prevent separation, and allow for efficient heat transfer during phase changes. Common PCMs used in emulsions include organic materials like paraffins, fatty acids, and esters, as well as inorganic salts and hydrated compounds. The idea is to utilize the latent heat of fusion of PCM to increase the thermal energy storage capacity of the base fluid. The total amount of heat stored by a PCM emulsion in a temperature range, *T_i_*–*T_f_*, in which PCMs undergo a phase change transition, can be obtained using the following equation:

$${∆h}_{totale,\;PCME}=\int_{T_{i}}^{T_{f}}c_{p}dT+∆h_{latent}$$

where $\int_{T_{i}}^{T_{f}}c_{p}dT$ is the sensible heat stored by continuous and dispersed phases due to the temperature difference, and ∆*h_latent_* is the latent heat associated with the phase change transition of PCM droplets. Figure 11 represents the heat storage capacity of a PCME.

Currently, some challenges hinder the large-scale exploitation of PCMEs, such as the colloidal instability due to thermodynamic reasons (coalescence, creaming, flocculation, etc.). Various papers have investigated strategies for improving stability or developing preparation methods that increase the emulsion stability with the solvent-assisted method [95] or micro- or nano-encapsulation [96,97].

**Figure 12:**
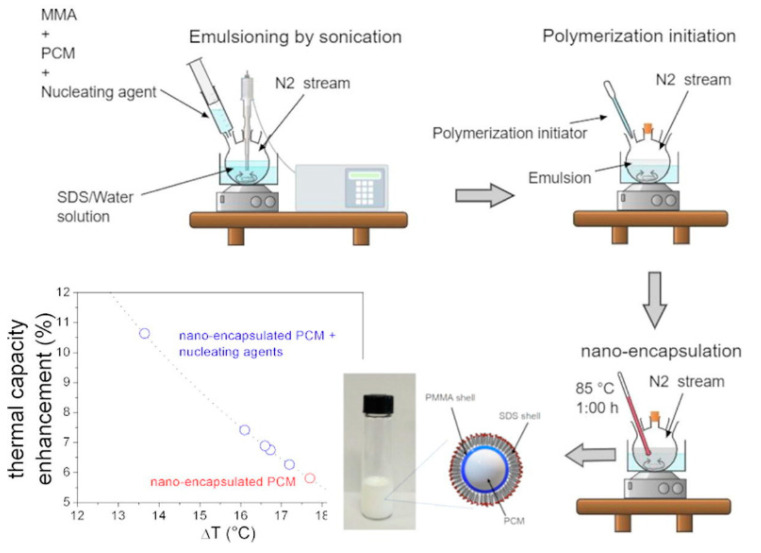
Example of PCME prepared by nano-encapsulating paraffin-based PCM in Poly(methyl-methacrylate) (PMMA) [97].

Figure 12 reports an example of a method for PCM nano-encapsulation in Poly(methyl-methacrylate) that we are investigating.

The applications of PCMEs in the energy sector are numerous, spanning solar–thermal and photovoltaic systems or solar–thermal–electric conversion [98,99,100], smart buildings [101], heat transfer and cooling [102,103,104], and thermal energy storage for district heating network [105].

**Figure 13:**
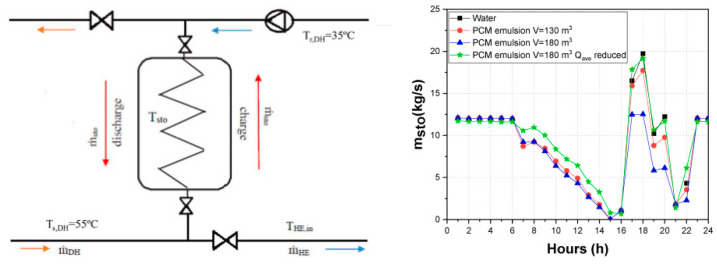
Decentralized thermal energy storage connection scheme with district heating grid (**left**) and storage mass flow comparison (**right**) from [105].

Figure 13 shows an example of the use of a cost-effective PCME in decentralized thermal energy storage for district heating network enlargement [106] with PCMEs accumulating a greater amount of heat than water in the same configuration of the system, generating a smoother operation with less abrupt changes in the circulating mass flow and a reduction in the storage volume and consequently in the investment costs.

**Figure 14:**
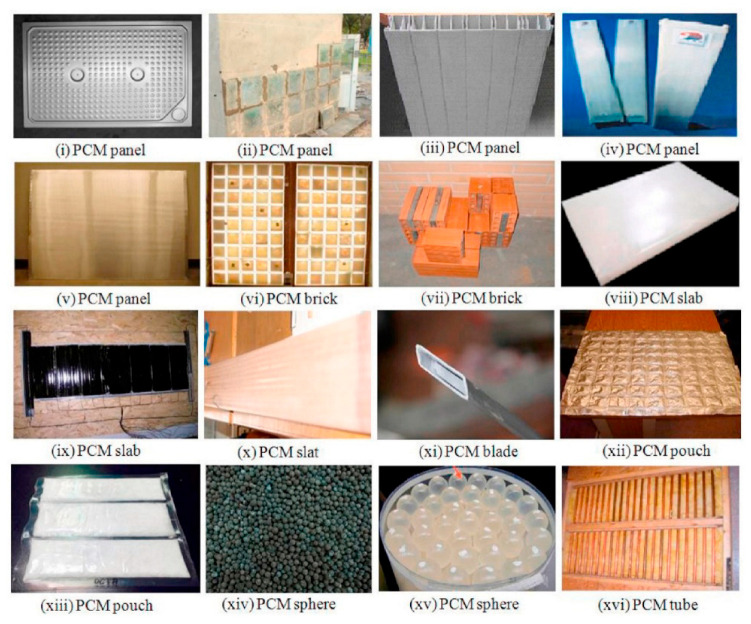
Different macro-encapsulation forms used in building structures from [108].

The application of LHTES in buildings has many advantages including the ability to narrow the gap between the peak and off-peak loads of energy demand, by storing solar energy during the day, and releasing it at night, thus reducing daytime temperature fluctuation, improving the degree of thermal comfort, and reducing cooling costs. In general, LHTES increases the thermal inertia of building components. Therefore, PCMEs have been incorporated into wall materials such as gypsum wallboards and concrete to enhance the thermal energy storage capacity of buildings with particular interest in passive solar applications, peak load shifting, etc. [106]. Other applications include thermally activated ceiling panels with macro-encapsulated PCMEs in lightweight and retrofitted buildings and in night ventilation [107]. Figure 14 reports some examples of macro-encapsulation forms of PCM used with building structures for thermal comfort and energy saving [108].

Some simulations evaluated the effects of the application of PCMs in different building positions on the energy consumption of a building in a hot-dry climatic region, always reducing energy consumption [109].

**Figure 15:**
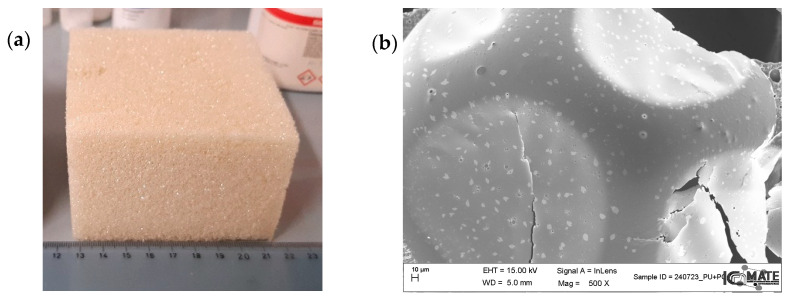
(**a**) Example of PU with 10 wt% of paraffin-based commercial PCM with heat storage capacity of 15–16 kJ/kg, developed in our ICMATE CNR laboratories. (**b**) SEM micrograph of PCM embedded into the PU matrix.

Concerning PCMs’ use in insulating materials, some studies have been conducted on the thermal characterization of PCM in polyurethane (PU), a commercial material used for insulating components [110]. In particular, a study tested a polyurethane thermal insulation panel with PCME, finding that PCME was able to impart good LHTES properties to the foams [111]. Figure 15 presents a picture of an example of polyurethane containing 10 wt% of a paraffin-based commercial PCM (Rubitherm, RT 28HC) that has a heat storage capacity of 15–16 kJ/kg (a), along with a SEM micrograph showing the PCM domains embedded into the PU polymeric matrix.

Some studies have also reported the investigation of PCM/geopolymer composites in construction materials [112], showing that different chemical compositions, gel structures, and texture properties affect energy storage capacity and kinetics.

Thus, thermal energy storage based on LHTES, and more specifically on PCMEs, offers the potential to optimize energy use in various sectors. PCMEs have shown significant potential in improving heat transfer in fluids for heating and cooling applications. Despite their benefits, PCMs face challenges that must be addressed for their widespread adoption related to (i) finding a proper way to disperse PCMs in geopolymers and to reduce composite costs, (ii) improving colloidal stability, (iii) addressing supercooling, and (iv) developing effective encapsulation techniques to contain leaks and improve thermal conductivity. As research and development continue to address challenges and explore new possibilities, PCM emulsions are poised to play a crucial role in achieving energy efficiency and sustainability in the years to come.

## 4. Thin Films for Hydrogen Energy Technologies

Scientific advances in materials play a key role in the transition toward hydrogen sustainable energy systems. Thin-film technologies offer a promising avenue for the development of efficient, cost-effective, and scalable hydrogen energy solutions. These films, characterized by their thickness on the nanometer to micrometer scale, have found applications across the entire hydrogen energy value chain, from production and storage to conversion and utilization. Here, we focus on our recent research on hydrogen barrier coatings and hydrogen permeation membranes that are expected to become crucial in promoting effective hydrogen storage and transportation in the future.

### 4.1. Nitride Thin Films as Hydrogen Permeation Barriers: An Advanced Solution for Hydrogen Embrittlement Prevention

In the hydrogen storage and transportation sector, the development of barrier systems for reducing hydrogen embrittlement (HE) of structural materials is becoming of paramount importance, which may compromise their mechanical integrity with increased susceptibility to cracking, severely jeopardizing structural integrity, especially in tanks and pipelines. Steel, aluminum, magnesium, and titanium alloys may experience HE, which could be mitigated by blocking or limiting hydrogen diffusion inside the material by applying suitable coatings, acting as hydrogen permeation barriers (HPBs). Concerning materials, polymeric films produced by thermal spraying are promising HPBs: even though polymers present poor mechanical strength, their low hydrogen permeability values are attractive [113]. Other extremely interesting materials for this application are ceramic coatings such as oxides, nitrides, and carbides [114]. Among them, nitrides, including boron nitride (BN) [115], silicon nitride (Si_3_N_4_) [116], and zirconium nitride (ZrN) [117], have been extensively explored for HPB purposes, due to their characteristics of high strength and thermal resistance. Chromium nitride (CrN_x_) is also commercially used as a permeation and corrosion barrier on stainless steel [118,119]. Such coatings exhibit a mixed CrN cubic and Cr_2_N hexagonal structure and have a low hydrogen permeability due to the higher packing density of the cubic phase [120]. Ternary nitrides, such as AlCrN and CrWN, are also considered as effective barriers against hydrogen permeation [121]. To address HE and related issues, titanium nitride (TiN) [122,123,124] together with its ternary compound aluminum–titanium nitride (TiAlN) and TiAl/TiAlN multilayer coatings have emerged as a promising solution, offering superior barrier properties that inhibit hydrogen penetration while maintaining desirable mechanical and thermal characteristics, due to the improved corrosion resistance conferred by aluminum [125]. The alternating TiAl/TiAlN layers create a barrier system that exploits the unique properties of each material. This configuration ensures that hydrogen atoms face multiple interfaces and diffusion paths, significantly hindering permeability. The multilayer design also improves adhesion and reduces coating defects, further enhancing efficacy.

Creating effective TiAl/TiAlN multilayer coatings requires precision engineering and advanced manufacturing techniques. Physical vapor deposition processes such as DC magnetron sputtering and high-power impulse magnetron sputtering (HiPIMS) [118] are widely used to produce nitride coatings. These methods ensure high purity and controlled layer thickness. HiPIMS represents one important improvement among the magnetron sputtering technologies [126]. Indeed, its highly dense plasma (up to 10^20^ m^−3^ near the target) enhances the electron-impact ionization of sputtered atoms, resulting in a significant fraction of ionized species. Applying a negative bias voltage to conductive substrates, the intense ion bombardment of the growing film improves adatom mobility, potentially leading to the synthesis of adherent, ultra-dense, smooth, defect-free, and uniform films, which are crucial properties for achieving suitable HPB layer performance.

**Figure 16:**
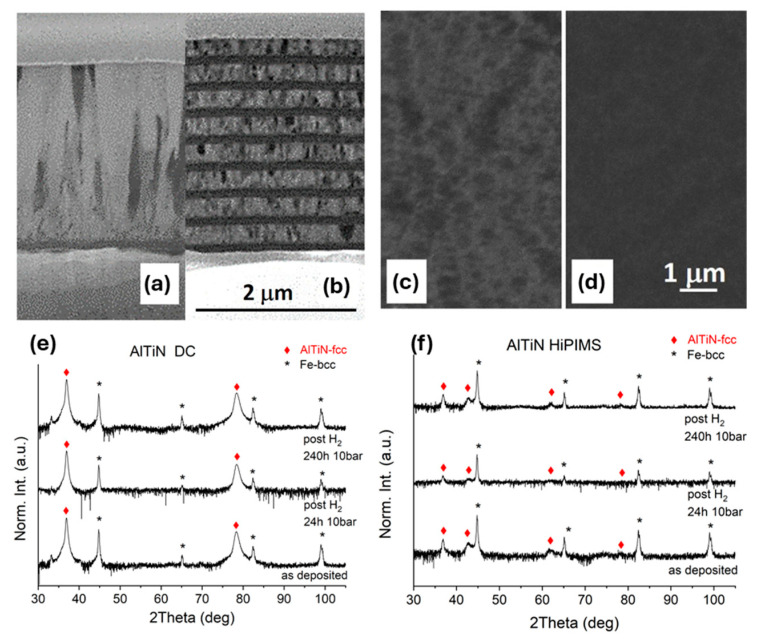
FIB cross-sections of two coatings grown by DCMS: (**a**) a bilayer and (**b**) a multilayer coating. Representative samples of TiAl/TiAlN films: SEM top views of two film deposited by (**c**) DCMS and (**d**) HiPIMS. XRD spectra of as-deposited and hydrogen-exposed films by (**e**) DCMS and (**f**) HiPIMS.

Controlling the microstructure of the multilayer system is essential to achieve the desired barrier properties. Parameters such as layer thickness, composition, and interface quality are carefully tailored. As an example, DCMS samples of FIB cross-sections of a TiAl-TiAlN bilayer (Figure 16a) and of a multilayer coating (Figure 16b) are also shown. The multilayer sample is produced by alternating metallic TiAl layers (100 ± 10 nm thick) and ceramic TiAlN films (220 ± 10 nm thick) that show an average lateral grain dimension of 62 ± 8 nm (image analyzed using the ImageJ 1.46r software). All metal (TiAl)–nitride (TiAlN) interface zones showed typical small, nearly equiaxed grains exhibiting a low pronounced columnar growth [127]. Concerning the bilayer case, the TiAlN film is about 2.3 μm thick, and the average lateral grain size is 150 ± 35 nm. By increasing the thickness, the small grains at the interface zone rapidly evolve into a competitive columnar growth (Figure 16a). Several studies report that, in polycrystalline materials with columnar grains oriented orthogonally to the substrate surface (Figure 16a), grain boundaries behave as diffusive channels for hydrogen, negatively affecting the barrier properties of the film [128]. Therefore, multilayer coatings composed of densely bound microcrystalline grains (Figure 16b) have been deposited to improve the hydrogen barrier performance of the system [129]. In TiAl/TiAlN multilayer coatings, defects and pores present in each single layer are interrupted at the interface by the next layer, further increasing the efficiency against HE [130,131]. The multilayer structure effectively blocks hydrogen diffusion pathways, reducing permeation rates. Figure 16c,d show the surface images of two representative samples of TiAl/TiAlN bilayer films obtained by DC magnetron sputtering and HiPIMS, respectively. Compared to the DC sample (Figure 16c), the HiPIMS nitride bilayer coating (Figure 16d) presents a very smooth and featureless surface, denoting a uniform and homogeneous deposition throughout the sample. TiAlN films exhibit remarkable stability under hydrogen exposure up to 10 bar for 240 h, with no observable microstructural degradation or secondary-phase formation (Figure 16e,f). These findings confirm the suitability of TiAlN as a robust hydrogen permeation barrier material.

Finally, in general, it must be considered that amorphous films have better barrier properties than crystalline ones. An additional improvement can be obtained by adding elements such as Si, which contribute to obtaining a quasi-amorphous film, thus exhibiting a low structural order at medium and long ranges. Despite their promising attributes, TiAl/TiAlN coatings face challenges such as cost, scalability, and optimization. Thus, research efforts are focused on improving their mechanical robustness and chemical stability. Furthermore, scaling up the production of high-quality multilayer thin films for industrial applications remains a challenge. As research progresses and manufacturing techniques improve, these coatings are poised to play an even greater role in ensuring the safety and longevity of hydrogen-exposed materials.

### 4.2. Metallic Thin Films as Separation Membranes for Hydrogen Energy

**Figure 17:**
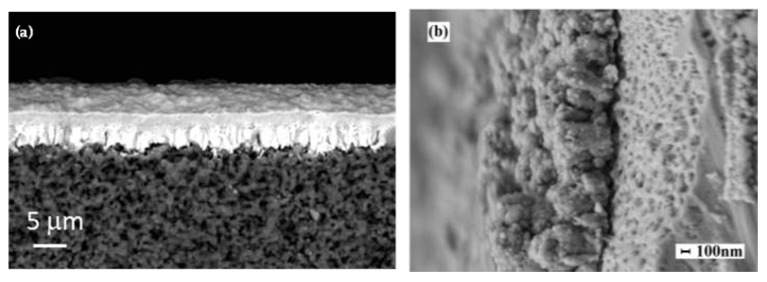
(**a**) PdAg film deposited by HiPIMS onto porous alumina and (**b**) PdAu film deposited by magnetron sputtering [135].

Hydrogen production from fossil fuels is mainly achieved through steam reforming, which generates a gaseous mixture rich in H_2_, CO, CO_2_, and other byproducts. The hydrogen must be purified for use in chemical production or fuel cells. Membrane technology is considered a viable alternative to traditional purification systems due to advantages such as low energy consumption, continuous separation, and ease of scalability [132]. Among metallic thin films, palladium-based membranes are considered the gold standard for hydrogen separation. Palladium (Pd) has a unique ability to absorb hydrogen molecules, dissociate them into hydrogen ions, and allow them to permeate through the membrane. This makes palladium highly effective in achieving high-purity hydrogen separation. However, challenges such as high cost, sensitivity to poisoning by sulfur compounds, and hydrogen embrittlement have led researchers to explore Pd alloys and alternative metals. Alloys such as palladium–silver and palladium–copper exhibit improved performance and reduced costs, making them more commercially viable. Another viable approach is to reduce the thickness of dense selective layers to films of a few µm, deposited onto porous substrates. Several membranes based on Pd and its alloys have been developed and tested as thin films on porous substrates such as alumina, nickel, or stainless steel [133]. Magnetron sputtering has proven to be an effective method for depositing these selective layers onto porous substrates [134,135]. In particular, high-power impulse magnetron sputtering (HiPIMS) resulted to be suitable for depositing a hydrogen-selective membrane PdAg film onto porous alumina [136]. Figure 17 reports few examples of palladium alloys deposited by magnetron sputtering for hydrogen separation applications.

To further reduce the use of critical raw materials and membrane costs, partial or total replacement of palladium is a prospect to be pursued, and much research is ongoing on this topic. Some group IV and V metals, such as vanadium, show high hydrogen permeability but, if uncontrolled, their huge solubility can compromise mechanical stability (embrittlement). Several vanadium-based alloys have been tested to substitute Pd-based membranes [137,138]. In this context, the magnetron sputtering technique has been widely used to deposit thin films of palladium or palladium alloy onto bulk metal membranes, with the dual function of avoiding oxidation and catalyzing hydrogen splitting [139,140]. In a few cases, thin films other than palladium were deposited by magnetron sputtering such as NbC [141], Ni_64_Zr_36_ [142], or Mo_2_N [143].

**Figure 18:**
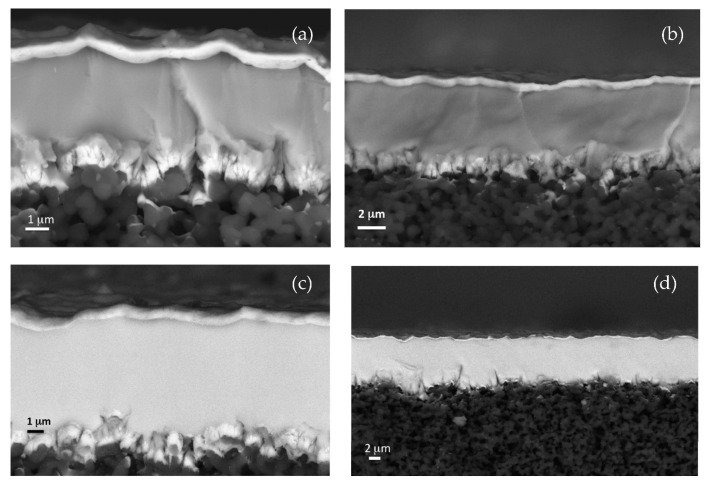
Some representative cross-sectional views of membranes based on various Pd/Zr_x_V_y_Ti_z_Pd_w_/Pd multilayers on porous alumina with varying compositions from only ZrVTi al loy (**a**) and with the addition of 19 (**b**), 23 (**c**) or 21 (**d**) at % of palladium [145].

The magnetron sputtering technique also enables the deposition of a selective non-noble layer and of a thin protective and catalytic film in a single vacuum stage, preventing oxidation at the interface. Therefore, some studies investigated the use of HiPIMS for the deposition of multilayers on top of porous substrates as membranes for hydrogen separation with a very low content of palladium. Some examples that have demonstrated suitable permeability and selectivity values, combined with resistance to hydrogen embrittlement, are Pd/V_93_Pd_7_/Pd multilayers on alumina substrate [144] or Pd/Zr_x_V_y_Ti_z_Pd_w_/Pd multilayers [145]. Figure 18 reports an example of cross-sectional views of these Pd/Zr_x_V_y_Ti_z_Pd_w_/Pd multilayers.

Thus, developing efficient and cost-effective methods for producing metallic thin films at scale is a critical area of research. Implementing surface modifications to improve resistance to poisoning and the embrittlement of these multicomponent layers make them a promising solution as membranes that play a pivotal role in the global transition to a hydrogen-based energy economy.

## 5. Ferroelectric Ceramics and Composites as Transformative Approach to Energy Storage and Harvesting

**Figure 19:**
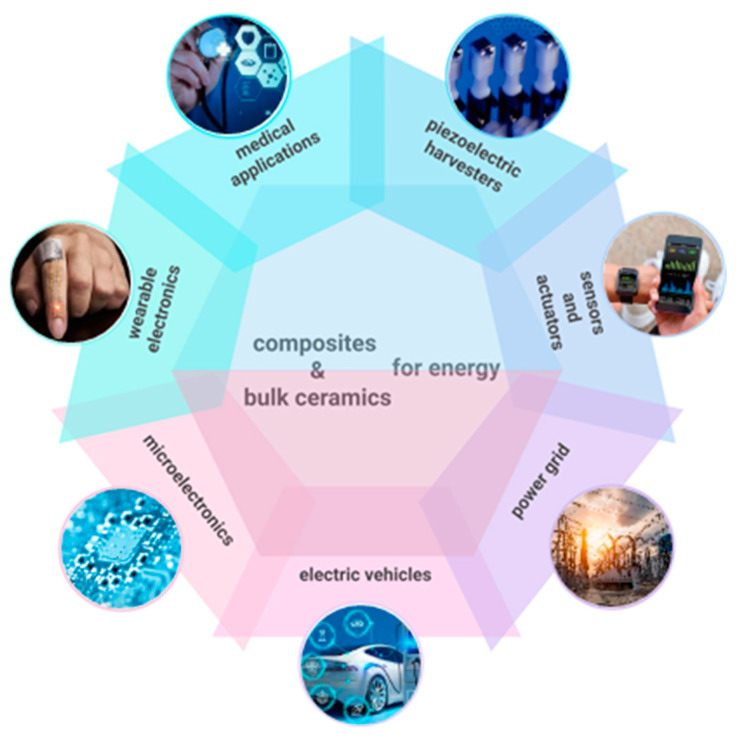
Different applications for energy of ferroelectric bulk ceramics and composites.

Electrostatic capacitors, particularly relaxor ferroelectric (RFE) ceramics, are one of the most common classes of energy storage systems due to their high-power density and rapid charge/discharge capabilities. Their potential for high-voltage applications and integration into portable electronics makes them attractive; however, enhancing their energy density remains crucial for broader applications, as shown in Figure 19. Chemical substitution is the primary method to tailor the functional properties of ceramics. Lead-free BaTiO_3_ (BT)-based systems have been widely investigated to understand the mechanisms behind relaxor behavior and how compositional tuning can optimize energy density, especially near the transition from classical ferroelectric (FE) to RFE. For homovalent Ti-site substitution (e.g., Sn^4+^, Hf^4+^, Zr^4+^, Ce^4+^), the degree of substitution governs the relaxor behavior [146,147,148,149]. For heterovalent substitution, the transition occurs at a much lower substitutional level, primarily due to charged defects [150].

By targeting the crossover between FE and RFE behavior, significant improvements in energy storage density have been achieved. Yuan et al. [151], using machine learning, reported a high energy storage density of ≈73 mJ cm^−3^ at 20 kV cm^−1^ with 90% efficiency for (Ba_0.86_Ca_0.14_)(Ti_0.79_Zr_0.11_Hf_0.10_)O_3_. Similarly, a more complex RFE solid solution, 0.9Ba_0.65_Sr_0.35_TiO_3_–0.1Bi(Mg_2/3_Nb_1/3_)O_3_, exhibited a high energy storage density of 3.90 J cm^−3^ and an ultrahigh recoverable energy storage (W_rec_) of 3.34 J cm^−3^ at 400 kV cm^−1^ [152].

To further enhance performance, especially breakdown strength (BDS), several strategies have been explored. As porosity and defects cause electrical failure, increasing ceramic density using sintering aids, core–shell particles, or composites with insulating phases like SiO_2_ or MgO can locally modulate the electric field and boost BDS [153]. Another approach involves increasing grain/grain boundary resistivity and reducing grain size by Ta_2_O_5_ excess, achieving W_rec_ = 9.03 J cm^−3^ and BDS = 720 kV cm^−1^ with 95% efficiency in 0.75BaTiO_3_–0.25Bi(M_1–0.015x_Ta_0.015x_)O_3+0.015x_ (M = Mg_2/3_Ta_1/3_) [145,154].

Recently, record performance was achieved using in situ phase separation in the composition (0.6–x)(Bi_0.5_Na_0.5_)TiO_3_–0.4BaTiO_3_–xCdZrO_3_ (x = 0.10), yielding W_rec_ = 23.6 J cm^−3^ at 99 kV mm^−1^ with high efficiency, due to the synergistic interaction of the two resulting phases [155].

Improving energy storage performance remains a key challenge for bulk perovskite ceramics and a number of strategies have been exploited. Trends toward replacing expensive noble metal electrodes (Pt-, Ag-based) with base metals (Ni, Cu) necessitate a deeper understanding of ceramic/electrode interfaces [156].

For flexible electronics, ceramics brittleness is a limitation. Polymer–ceramic composites offer a solution by combining the flexibility, durability, and high BDS (1500–5000 kV cm^−1^) of polymers with the high permittivity of FE ceramics. These materials are ideal for high-energy density capacitors, actuators, and sensors [157].

Lead-free ceramics like BT, (Bi_0.5_Na_0.5_)TiO_3_–BaTiO_3_ (NBT–BT), and Ba(1–x)Ca_x_Zr_γ_Ti₍_1–γ_₎O_3_ (BCZT), with strong piezoelectric properties, are preferred over toxic lead zirconate titanate [158]. Among polymer matrices, PVDF and its copolymers are widely studied for their high dielectric constant (~10), piezoelectric response (d_33_ > 20 pC N^−1^), low dielectric loss, and exceptional BDS, much higher than that of pure BT (~100 kV cm^−1^) [151].

The performance of polymer–ceramic composites can be tailored to specific applications through microstructure engineering, primarily via the careful selection of filler composition, morphology, and surface modification (e.g., core–shell structures or functionalization) [159,160]. Fabrication techniques like melt blending, spin coating, shear-induced alignment, and field-assisted processing further enhance particle dispersion and orientation, optimizing energy density or piezoelectric response [161].

In these composites, permittivity generally increases with the volume fraction of inorganic fillers. For example, adding 30 vol.% BT can yield permittivity values between 20 and 40, depending on processing, particles synthesis, and functionalization methods [162]. Piezoelectric performance also scales with the filler content: Craciun et al. showed that increasing the NBT-BT content in PVDF from 40% to 50% raised the piezoelectric constant (d_33_) from 13.5 to 33 pC N^−1^ (at 2 MHz), compared to 0.2 pC N^−1^ in pure PVDF [163]. However, excessive filler loading can cause higher dielectric losses and mechanical degradation, possibly reaching the percolation threshold with a reduction in overall efficiency [164].

Particle size and dispersion are critical. Small particles (~100 nm) enhance interfacial interactions and polarization but often agglomerate, while larger particles (>1 μm) may improve dielectric properties at the cost of flexibility and processability [165]. Regarding composite architecture, 0-3 structures (randomly dispersed fillers in a polymer) are easier to fabricate but face issues like poor stress transfer, aggregation, and inefficient poling. In contrast, 3-3 structures (interconnected ceramic and polymer phases) offer superior field distribution and stress transfer, significantly improving power generation, though they are complex and expensive to fabricate [166].

Filler morphology also plays a key role. Huang et al. demonstrated that composites containing BT nanofibers achieved higher breakdown strength (BDS = 691.9 kV mm^−1^) and 45% greater energy density than those with spherical particles (BDS = 585.5 kV mm^−1^) [167]. Numerical studies further confirm that continuous fiber fillers yield higher strain and electrical energy densities than short fibers or spheres [155]. However, particle aggregation or contact between fillers can enhance local electric fields—especially at neck regions—reducing BDS [165].

**Figure 20:**
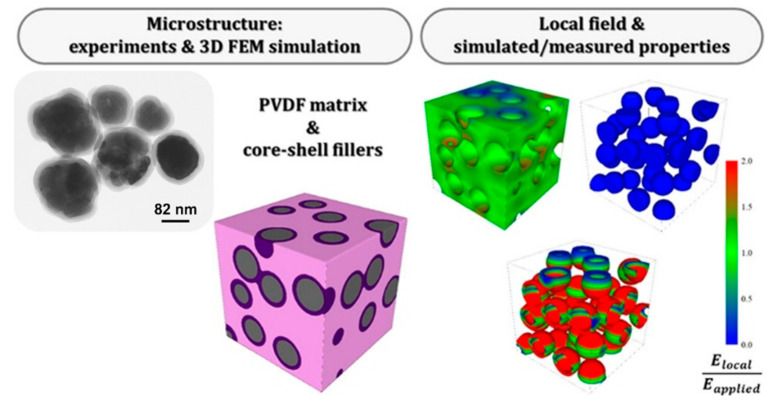
TEM image of core–shell BT particles and local electric field maps and distributions as determined by FEM simulations.

To address this, Padurariu et al. [168] used 3D FEM simulations to propose insulating oxide (SiO_2_ or TiO_2_) coatings on BT fillers (Figure 20). This core–shell design lowered dielectric constant (to 32 at 1 kHz) but significantly reduced dielectric loss (<0.03 across 10^2^–10^4^ Hz) and improved high-field endurance. Such coatings offer protective effects, enabling higher BDS, as also shown in ceramic systems [154].

Enhancing the piezoelectric performance of polymer–ceramic composites can be effectively achieved through processing techniques such as electrospinning and patterning, which enable precise control over the alignment of fillers and polymer chains ([169] and references therein). Aligned anisotropic ceramic fillers (e.g., BT nanorods, fibers, sheets) facilitate better dipole orientation, improving charge transport and effective permittivity and reducing dielectric losses—resulting in higher d_33_ values and energy conversion efficiency [170].

Texturing strategies for lead-free materials aimed at flexible energy harvesting and sensing applications have also been demonstrated [171]. Aligned MWCNT-based composites show enhanced strain sensitivity and mechanical properties due to more efficient electrical networks and load transfer, compared to randomly dispersed fillers [172].

A notable example is the work of Athira et al. [173], where a flexible nanogenerator based on electrospun PVDF–BT nanofibers achieved a power density of ~4.07 mW m^−2^, thanks to interfacial effects that increased the polar β-phase of PVDF to ~91%. The device served effectively as a self-powered vibrational sensor.

Additionally, 3D printing has emerged as an advanced fabrication method that allows accurate control over composite architecture and filler distribution, fostering innovation in composite performance, sustainability, and material design [174].

Ongoing research focuses on improving the understanding of ceramic–polymer interfacial phenomena and clarifying the interdependence between composition, microstructure, and properties to unlock further performance gains in these multifunctional materials.

## 6. Metallic Materials for Energy Applications

Metallic materials are indispensable in a wide array of energy-related applications due to their unique properties, such as high thermal and electrical conductivity, mechanical strength, and corrosion resistance. These materials are integral to conventional energy systems, including fossil fuels and nuclear power, as well as emerging renewable energy technologies, such as wind, solar, and hydrogen energy. The selection, design, and optimization of metallic materials are critical to improving the efficiency, longevity, and sustainability of these energy systems, and some examples are discussed below.

### 6.1. Cu-Based Alloys Manufactured by Additive Manufacturing for Advanced Heat Exchangers

**Figure 21:**
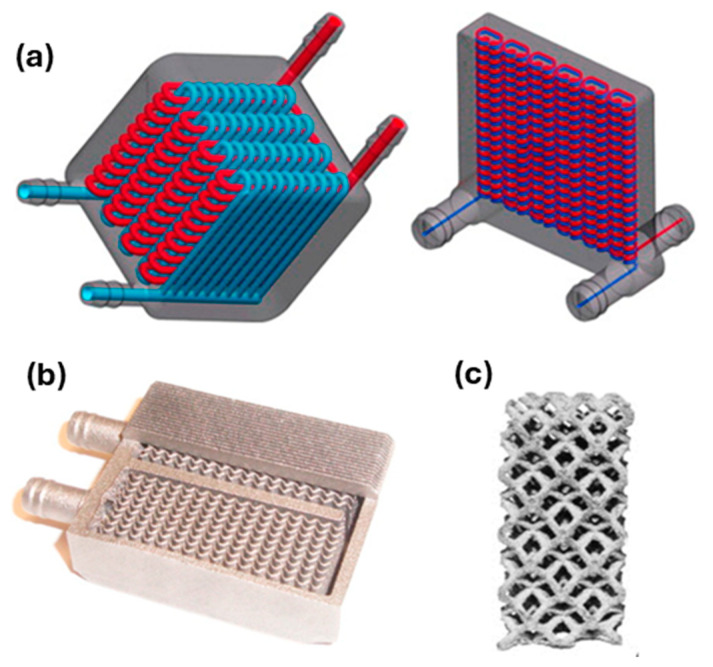
Examples of heat exchanger components (**a**–**c**) of different geometry and periodicity manufactured using AM technologies with Cu alloys [177].

Metals such as copper and aluminum exhibit excellent thermal conductivity, facilitating heat transfer in systems like power plants and heat exchangers. Additive manufacturing (AM) is an advanced manufacturing method, which allows the achievement of 3D complex structures through a layer-by-layer strategy. Among AM technologies, the most widespread one in the industrial environment is Laser Powder Bed Fusion (LPBF), which relies on a laser beam scanning a powder bed for the material consolidation. In the energy field, Ni alloys, such as Inconel 625 and 718, and Al ones, such as AlSi10Mg, are well-consolidated materials to be fabricated using the LPBF process, while nowadays the most novel achievements are oriented to the development of novel structures in pure Cu and Cu-based alloys for thermal management systems. In this case, the design for AM can offer unique opportunities for producing high-performance heat exchangers, thanks to the high degree of geometrical complexity as well as the high surface-to-volume ratio that can be reached by adopting AM production [175]. For instance, AM can promote the use of advanced devices dedicated to heat transfer, like radiators, coolers, exchangers, induction heat coils, and radio frequency cathodes that are able to gain an increase in their functional performances, thanks to the high surface-to-volume ratio and reliability, due to the lack of soldering or welding processes [176]. Figure 21 shows some representative examples of heat exchanger components produced by AM with Cu-based alloys.

Laser material processing, including LPBF, of pure Cu and Cu-based alloys represents a considerable technological challenge, due to the low absorption coefficient of such alloys under laser irradiation as well as their high thermal conductivity. To tackle such limitations, two main strategies are investigated, i.e., (i) the development of Cu alloys with novel chemical compositions, with the main scope of improving the LPBF processability for high-quality parts, and (ii) the exploration of different laser sources, emitting the radiation far from the infrared (IR) field, which is typically used for processing metals, but is not able to offer acceptable absorption values for highly reflective materials. For instance, CuZrCr and CuCrNb alloys are promising for their good balance between processability and functional properties.

**Figure 22:**
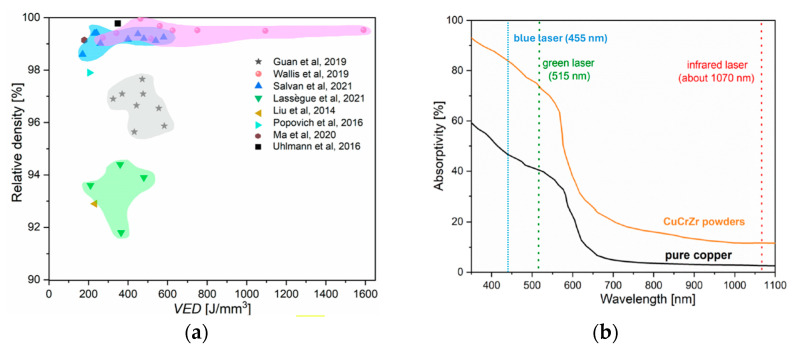
(**a**) Representative LPBF feasibility map of CuCrZr powder showing trend of relative density versus energy. (**b**) Absorptivity versus emission wavelength for pure Cu and CuCrZr alloy. Three vertical lines represent characteristic emission wavelengths of blue, green, and infrared lasers; the references mentioned in the left figure can be found in ref. [177].

Figure 22a shows the processability map of CuCrZr powders, processed by using LPBF [178]: it shows that the achievable relative density of built parts can be as high as 99.5%.

The most recognized approach is oriented to the use of green or blue lasers that exhibit a high absorption for highly reflective materials [178,179,180], as shown in Figure 22b.

**Figure 23:**
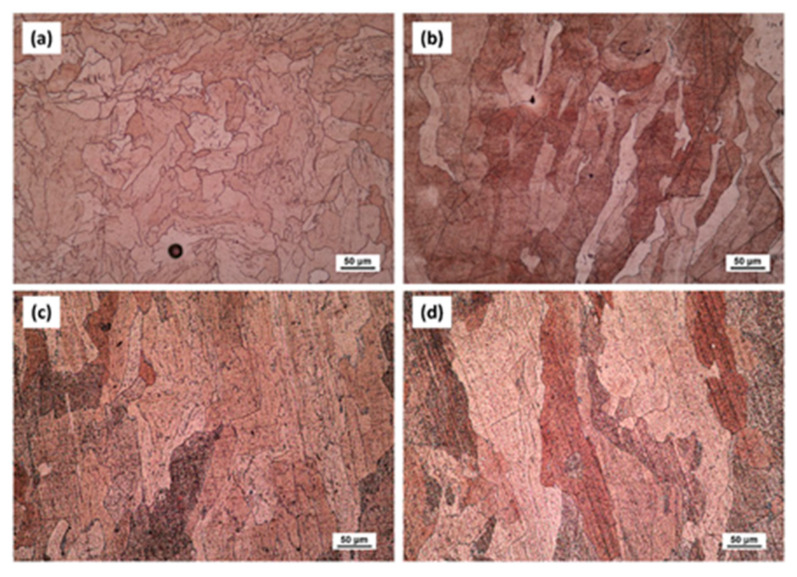
Microstructures of LPBFed CuCrZr in as-built (**a**,**b**) and heat-treated (**c**,**d**) conditions. Views on left show sections parallel to platform while those on right show sections along building direction [181].

The microstructure of LPBFed (Laser Powder Bed Fusion) Cu alloys typically exhibits fine grains due to the rapid solidification induced by the laser process. In the as-built condition, the grains are oriented along the building direction, which can be observed in the microstructure images in Figure 23 [181]. Post-building heat treatments are often required to relieve residual stresses and adjust the final properties, which can lead to grain growth and a more homogeneous microstructure.

**Figure 24:**
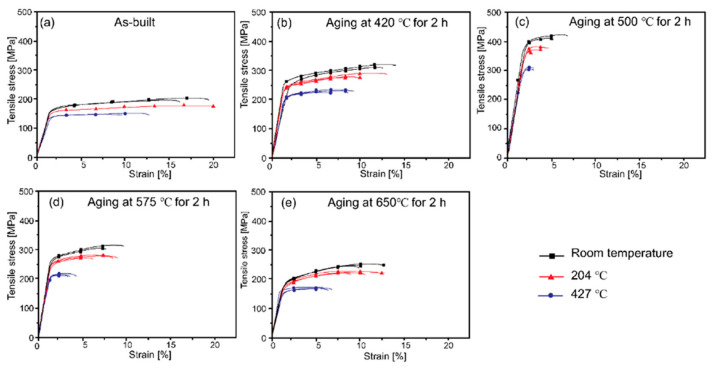
Evolution of tensile properties at varying testing temperatures and heat treatment conditions [182,183].

The mechanical properties of LPBFed Cu alloys can be tuned by heat treatments, which promote precipitation phenomena that strengthen the material, as shown in Figure 24.

**Figure 25:**
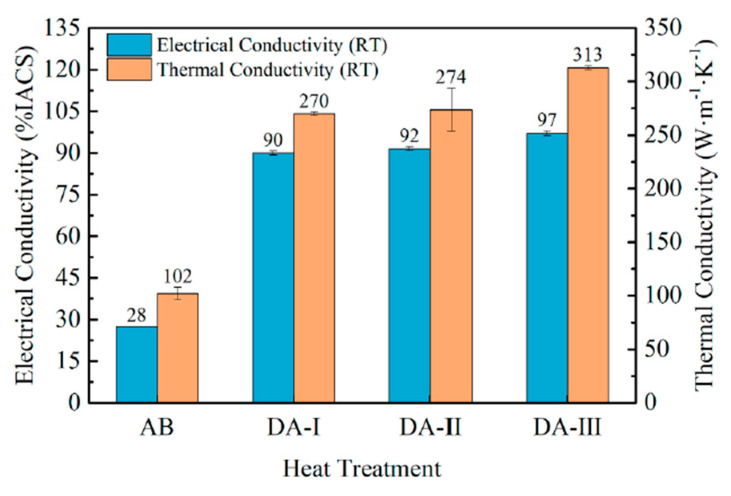
Electrical and thermal conductivity versus different heat treatment conditions [185].

Figure 25 shows the increase in both thermal and electrical conductivity by heat treatment [184].

The quality of manufactured parts significantly influences both mechanical and thermal/electrical performance, contingent upon the presence of residual pores and cracks. Consequently, the manufacturing process plays a critical role in determining the final performance of the components. It can be inferred that the in-service performance of LPBFed copper heat exchangers can be markedly enhanced by the design flexibility afforded by the additive manufacturing (AM) process and the selection of appropriate post-processing thermal treatments. Specifically, aging treatments offer a synergistic enhancement of both mechanical strength, due to the precipitation of secondary phases, and thermal conductivity, owing to the depletion of alloying elements from the copper matrix. Future research should focus on the development of novel alloys based on elements with low solubility/miscibility relative to the face-centered cubic copper phase.

### 6.2. Ductile Iron Castings as an Opportunity for Wind Energy

Wind energy represents a renewable, reliable, and environmentally sustainable power source that is rapidly expanding to mitigate CO_2_ emissions and combat climate change. Wind turbines, the pivotal components of this green revolution, necessitate materials capable of withstanding extreme conditions, providing structural integrity, and ensuring prolonged service life. Among the materials that show significant promise for the industry, ductile iron (DI) castings emerge as a noteworthy option. Renowned for their strength, flexibility, and cost-effectiveness, ductile iron castings are increasingly utilized in the wind energy sector. DI castings are the primary candidates for components such as main bearing housings, rotor hubs, and gearbox casings that demand high strength, durability, and resistance to dynamic loads. The capacity of DIs to dampen vibrations and endure harsh environmental conditions, such as those encountered in offshore wind turbines and hydroelectric plants, ensures the long-term reliability and performance of wind turbines. Furthermore, the scalability of DI components is a significant advantage when considering the upscaling of wind turbines. DI is a more economical alternative to cast steel and offers an extended service life due to its robustness and ease of maintenance [185,186].

Its durability results in fewer replacements and repairs, thereby reducing overall expenses and enhancing sustainability. Additionally, DI is fully recyclable, promoting further sustainability by minimizing raw material usage and the carbon footprint of production. Table 3 presents the major DI components employed in the construction of wind power plants and the average weight of the parts per megawatt. Despite its advantages, the application of DI castings in wind energy is not without challenges. For instance, the material’s density can increase the weight of components, which may be a disadvantage for certain applications. Moreover, achieving the precise mechanical properties required for wind turbine components necessitates stringent quality control during the casting process. Table 4 lists the minimum mechanical properties required for wind mill castings to comply with European legislation. The high ductility and toughness are of paramount importance for these castings due to the harsh weather conditions to which they may be exposed. Thus, the mechanical properties required for DI castings are crucial for the reliable and sustainable operation of wind power plants.

Material Quality Indexes (MQIs) for foundry products are designed to classify materials and their integrity, and they should be obtained from the basic tensile mechanical properties, because tensile testing is the most used and immediate testing method to characterize materials. About cast irons, one of the first MQI proposed by Siefer and Ortis and later developed by Crews in 1974 [187] is defined as MQI = R_m_^2^·A5, where Rm is the ultimate tensile strength and A5 is the elongation to rupture. Zanardi et al. [188] later refined this to MQI = R_m_^2^·A5/(8200 + 3R_m_). Based on this equation, for instance, new-generation spheroidal ductile irons, such as Isothermal Ductile Irons (IDIs) and Austempered Ductile Irons (ADIs), have MQI values over 360, while conventional spheroidal ductile irons with ferritic, ferritic–pearlitic, and pearlitic matrices range from 100 to 190.

However, an effective microstructure quality assessment should uniquely classify the matrix grade (ideal microstructure) based on composition and production route—limitations that MQI-based methods do not address. Indeed, an innovative mathematic procedure using Voce equation-based diagrams [189,190] has been recently developed by us that offers a clearer classification of different DI grades and their integrity [191,192].

Considering the relevance of mechanical behavior of DIs, we focus herein on innovative mathematical methods to correlate mechanical behavior with defects. This helps develop process–microstructure–properties relationships to assess from one side material integrity and the production routes of these materials, thus reducing the waste production, and from another side, to predict the lifetime of DIs, contributing to more sustainable, long-lasting solutions in the renewable energy production. The proposed method to assess the integrity of DIs focuses on analyzing tensile flow curves with the dislocation-density-related Voce equation [180,181], which is a simple, cost-effective alternative to complex and time-consuming defect detection techniques, such as metallography, non-destructive inspections techniques, or computer micro-tomography. The Voce equation is as follows:

*σ* = *σ*_v_ + (*σ*_o_ − *σ*_V_)∙exp (−*ε*_p_/*ε*_c_),

It is an exponential decay equation, where *σ* and *ε*_p_ are the true stress and plastic strain, respectively, while *σ*_V_ is the saturation stress, *ε*_c_ is the characteristic strain that defines the rate with which the *σ*_V_ is achieved, and *σ*_o_ is the back-extrapolated stress to zero strain. The differential form of Voce equation is as follows:

d*σ*/d*ε*_p_ = *Θ*_o_ – *σ*/*ε*_c_,

It can be used to investigate the strain-hardening behavior of materials. *Θ*_o_ is a constant that is related to the material microstructure, 1/*ε*_c_ is thermally activated dynamic recovery term, and the other parameters have the usual meaning. Strain-hardening parameters, *Θ*_o_ and 1/*ε*_c_, are sensitive to the microstructure and to materials’ defectiveness, so they can be used to assess the integrity of materials. In fact, if the Voce parameters *Θ*_o_ and 1/*ε*_c_, obtained from fitting the experimental strain-hardening analysis into the differential form of the Voce equation, are plotted in a Matrix Assessment Diagram (MAD), they lie on straight lines for both sound and defective castings. Interestingly, in sound castings, the intercept of the best-fit line is positive, while it is negative in defective ones. The second diagram is called the Integrity Assessment Diagram (IAD), which is created by plotting the experimental elongations to rupture versus the theoretical uniform elongations (where localized deformation occurs) calculated with the Voce formalism IAD can identify potential defects, as materials with defects rupture prematurely before the tensile localized deformation (necking), while sound materials rupture after necking.

**Figure 26:**
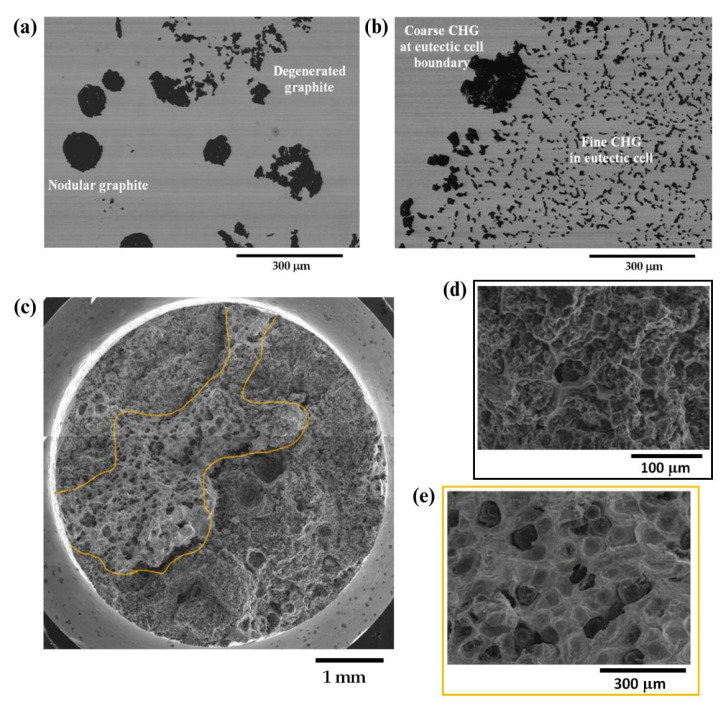
(**a**,**b**) SEM micrographs of polished section of tensile specimen showing graphite microstructure in GJS400_P casting; (**c**) general view of fracture surface with ferritic region and nodules (underlined in yellow) and regions covered in degenerated chunky graphite (CHG); (**d**) details at higher magnifications of regions covered in CHG; (**e**) regions with a ferritic matrix and nodules.

**Figure 27:**
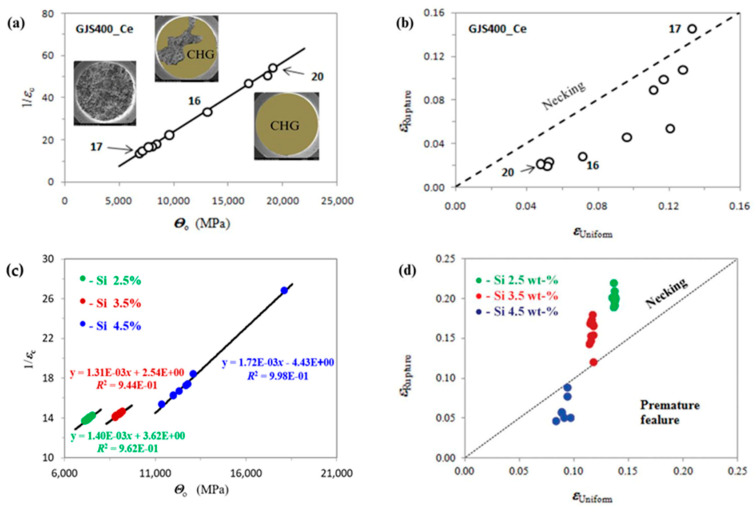
GJS400_Ce: (**a**) MAD; (**b**) IAD; (**c**) MAD for DIs with different silicon contents in % wt; (**d**) IAD for DIs with different silicon contents (% wt) produced in 50–75 mm Y-blocks.

In DI castings, defects like degenerated graphite, namely exploded, spiky, or chunky graphite (CHG), inclusions, gas, and shrinkage porosity can severely impact the integrity of the cast components and reduce their mechanical properties, including tensile strength, ductility, fatigue resistance, and fracture toughness. In heavy section components, like the ones used in wind turbines, degenerated graphite can be even more dramatic, significantly affecting their performance in the application. Therefore, production control and the establishment of new reliable procedures for integrity assessment of heavy sections are even more critical. An example of nodular and degenerated chunky graphite observed on the polished section and fracture surface of a GJS400 cast can be seen in Figure 26. As mentioned earlier, assessment diagrams such as MAD and IAD can provide information about the defectiveness of DIs. For instance, a ferritic spheroidal cast iron GJS400 with the addition of Ce to induce intentional degeneration of graphite into CHG was investigated, and it was demonstrated that the position of the Voce parameters in the linear dataset in MAD was related to the defectiveness of the tensile sample. In fact, the lowest Voce parameters values corresponded to the least defective microstructure (Figure 27a, Nr 17, showing a fracture surface with ferrite, nodular agglomerates and no CHG), with the longest elongation (17.85%) to rupture and the highest UTS (461.4 MPa), while the highest positions corresponded to the most defective microstructure (Figure 27a, Nr 20, a fracture surface completely covered in CHG, colored in brown), with the shortest elongation (2.53%) and the lowest UTS (391.9 MPa). Also, the IAD in Figure 27b shows that sample Nr 20 is the lowest data point in the plot, while sample Nr 17 has the highest position and experiences rupture after necking, indicating a sound microstructure without any CHG.

Furthermore, the use of MAD as an integrity assessment tool has been validated to effectively classify DIs by the silicon content, namely 2.5%, 3.5%, and 4.5% Si. With the silicon content higher than 3.0% wt, high-silicon strengthened ductile irons (HSSDI) are fully ferritic, making them cost-effective due to the elimination of heat treatment processes. Additionally, they are characterized by a narrow hardness range, which enhances their machinability. Figure 27c shows that as Si increased, the intercept of the best fitting line decreased, reaching −4.43 at 4.5% Si, indicating a defective microstructure in agreement with the low nodularity and the presence of CHG found in this composition. Moreover, the IAD analysis (Figure 27d) showed that the 4.5% wt silicon content led to reduced ductility and premature failure before necking in 50–75 mm Y-block samples, caused by graphite degeneracy. In contrast, samples from 25 mm Y-blocks and Lynchburg, with higher solidification rates, did not fail prematurely, though some ductility loss occurred due to the chemical ordering embrittlement effect.

**Figure 28:**
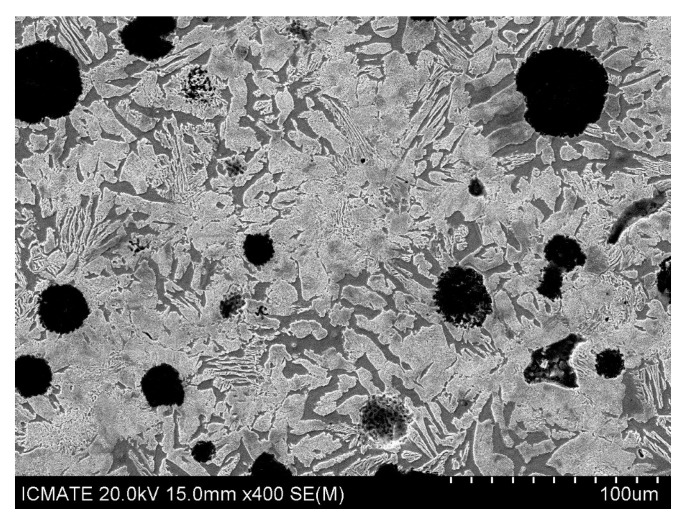
SEM micrograph with secondary electron signal of typical spheroidal graphite perferritic microstructure of IDI 800 after metallographic polishing and etching with 2% Nital, consisting of nodular graphite (black) embedded in alternated ferrite (gray) and pearlite (bright).

The same approach has also been used for innovative IDIs [193], namely IDI 800, that belong to a new generation of ductile irons that are very attractive due to the good combination of high mechanical properties and low production costs. IDIs are produced through heat treatment from conventional castings with a low content of alloying elements, e.g., typically GJS400 (C approximately 3.6% by weight, Si 2.46–2.66, Mn 0.10–0.15, Cu 0.01–0.15, Ni < 0.06, Mo < 0.01, and Sn < 0.01, equilibrium Fe). The low alloy content provides excellent mechanical properties even for heavy sections, since it results in few, non-dramatic segregation forms, making them particularly attractive for wind turbine components. Then, from room temperature, the cast iron is heated in the intercritical (a + g + graphite) range to be partially austenitized, leaving an appropriate fraction of proeutectoid ferrite. After quenching in a salt bath above the M_S_ temperature, the austenite is transformed into pearlite, resulting in a microstructure called perferrite that consists of alternating and interconnected regions of ferrite and pearlite, a structure that is different from the typical bull’s eye structure of conventional pearlitic–ferritic ductile irons, where the ferrite is surrounded by pearlite. Figure 28 illustrates an example of perferrite, where pearlite appears bright (typically exceeding 60% of volume fraction), ferrite is gray, and graphite is black (with a volume fraction of approximately 10%). Perferrite has excellent elongations at rupture: for instance, the minimum ductility of IDI800 is 6%, while it is 2.5% for conventional pearlitic–ferritic grades with the same UTS of 800 MPa. As seen in the IAD of IDI 800, almost 60% of the data points are above or slightly below the dichotomy line of necking, indicating the soundness of this novel IDI grade, while conventional pearlitic cast irons with similar UTS of 800 MPa fracture are by far more premature, i.e., before necking.

Thus, the studies conducted on ferritic spheroidal ductile irons (DIs), high-strength spheroidal ductile irons (HSSDIs), and innovative ductile irons (IDIs) have demonstrated the efficacy of Voce-based analysis as a robust assessment tool for evaluating the integrity and quality of castings. This analytical approach facilitates the production of cast components with enhanced durability, thereby promoting a sustainable methodology within the wind energy sector. The utilization of IDIs, characterized by their amalgamation of strength, flexibility, and economic viability, is pivotal as the global energy landscape transitions towards greener solutions. Therefore, we envisage the integration of such innovative materials as essential for ensuring the efficiency, longevity, and sustainability of wind energy systems.

### 6.3. Relevance of Material Design for Power Generation—Case Study of Ni-Based Alloys by Casting

In most power plants, fuel is burned to generate heat to drive steam for turbines. The conditions in boiler steam turbines and heat exchangers are relatively harsh, and the selected materials need to have the characteristics of heat resistance, high temperature resistance, and high creep resistance. A large number of favorable combinations of properties, such as excellent high-temperature mechanical properties, good oxidation and corrosion resistance, and ability to withstand extreme temperatures and high stress levels, make Ni-based alloys particularly suitable for power generation [194,195,196,197]. These alloys respond to the need of power generation covering hydraulic, thermal, nuclear, and wind energy production. Enhanced corrosion and erosion resistance of Ni- and Ni-Cr-containing alloys make them suitable for hydraulic turbines and electric generators, while Ni-alloyed steels are structural materials for wind turbines and solar collectors and pipes. Ni-based superalloys exhibiting high creep resistance and good corrosion are widely used to produce boilers and heat exchangers, as the parts of a power plant [193,198,199,200]. The most highly heat- and corrosion-resistant Ni-based alloys containing Fe and Cr are used as the parts of nuclear power plants, ensuring their long-term performance [201,202]. In different types of fuel cells, Ni and NiAl3 Raney alloy find applications as catalysts at the micro- and nanoscale [203,204]. Ni-based binary alloys containing Cr, Co, Mn, Cu, and Al are used in concentrated solar power (CSP) plants [205]; Ni-Mo, Ni-Co, and Ni-Fe-Co alloys as electrode materials for green hydrogen production [206]; and Ni-Mn-Co alloys for various types of rechargeable batteries [207]. In a broad context of the abovementioned applications, power generation is the industrial sector with a major contribution to greenhouse gas emissions. Therefore, questions are raised about environmental sustainability of Ni-based alloys, from ore extraction and metal processing to the impact of products in use and their recycling at the end of life, aiming to reduce the levels of pollutants to their permissible limit or even to eliminate them [208].

During the last sixty years, different types of Ni-based superalloys have been developed and grouped into series based on their composition, such as NIMONIC, INCONEL, HASTELLOY, CMSX, RENÉ, TMS, MC, INCOLOY, etc. [209,210]. Ni-based superalloys contain ten or more metallic elements, such as Ni, Co, Cr, Mo, Ta, Ti, Re, Nb, Hf, W, Al, and Fe alloyed with non-metals such as B, C, P, and Si, usually added into the base alloys as trace elements acting as grain refiners or for reducing grain defects. In Ni-based superalloys, the *AlNi*3 (*γ*′) intermetallic phase exhibits a high degree of order at high temperatures, and it is a key to strengthening in this class of alloys. Various combinations of Co, Mo, Ta, W, Nb, and Re in complex Ni-based alloys provide the highest level of strengthening observed to date.

**Figure 29:**
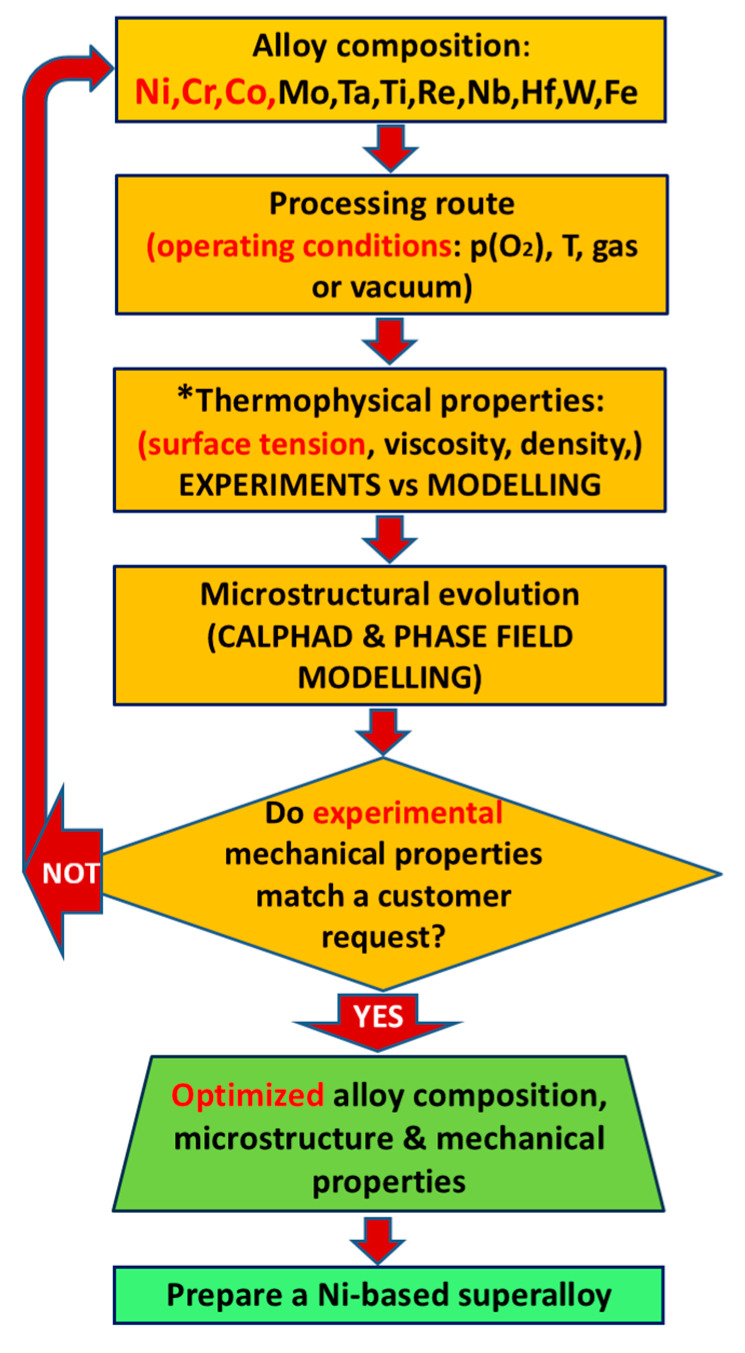
Flowchart summarizing steps of Ni-based alloy design by casting. (*) Key properties for modelling.

The most common approach to achieve a broad range of applications in materials science with high performance levels is material design. Its methodology involves a series of steps, iterations, and periodic feedbacks between theory, experiment, and computations, making it possible to perform investigation based on a quadriade “composition–processing–microstructure–properties” [211,212]. Among the processing routes used for manufacturing of Ni-based industrial alloys, an investment casting as one of the oldest known metal-forming techniques is still under continuous optimization, aiming to reduce the presence of structural defects on the components made from these alloys [213]. To this end, a particular attention must be paid to the simulation of the solidification in terms of microstructural evolution that requires sophisticated methods such as phase field, direct dendritic needle networks, finite element, etc. able to solve interfacial problems during solidification [214,215]. To predict accurately the spatiotemporal evolution of microstructures during solidification, the models describing thermodynamic stability, transport, and micromechanical response are available from different material disciplines, i.e., thermodynamics and CALPHAD (Calculation of Phase Diagrams)-type modeling including databases; kinetics (diffusion and convection models); and mechanics (elasticity and plasticity models) together with the accurate and reliable thermophysical properties data (surface tension, viscosity, diffusivity, density, specific heat, etc.) as inputs. Recently, combining theories with experiments, an advanced method of product design known as digital twin was applied to virtually replicate the processing of single-crystal Ni-based superalloy turbine blades using directional solidification inside a casting component. An example of a knowledge-guided alloy design to develop novel Ni-based superalloys is shown in Figure 29.

In alloy design, the role of the CALPHAD method is twofold: for the calculations of thermodynamic functions of mixing (the enthalpy, activity, integral and partial Gibbs energies) as well as for the assessment of phase diagrams aimed to determine thermodynamic stability of a system by means of its lowest energy or chemical equilibrium with its environment and/or be used as the part of models describing the thermophysical properties like the surface tension and viscosity.

Therefore, models predicting the thermophysical properties of Ni-based alloys as functions of composition have been developed in the framework of thermodynamics, statistical mechanics, and hard sphere-like theory. The preliminary analysis of the experimental data on the surface tension and viscosity, used to validate the existing models, indicates that the surface properties of Ni-based alloys can be properly described using the compound formation model (CFM) [216], while for the viscosity, the results of the Terzieff model are the most appropriate [217]. However, until now, no theory exists which describes satisfactorily the surface tension and viscosity of multicomponent alloys. In the case of liquid Ni-based complex alloys, both properties were evaluated with respect to those of Ni-Al alloys and taking into account the effects of the minority alloying elements.

**Figure 30:**
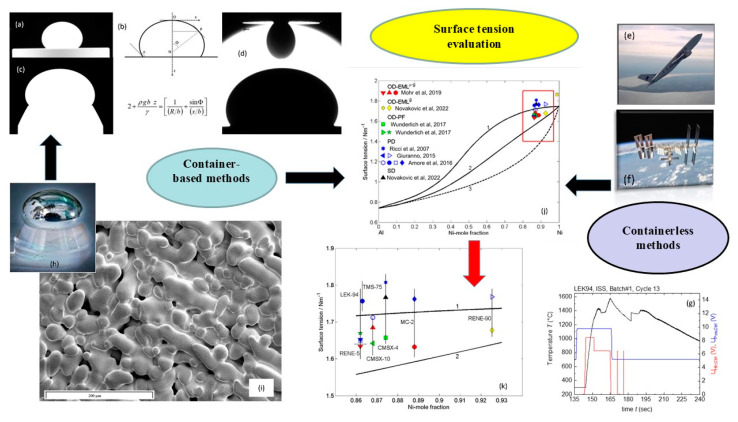
Surface tension experimental data of liquid Ni-based superalloys obtained by container based methods: (**a**) sessile drop (SD); (**b**) equilibrium drop profile described by fitting equation; (**c**) large drop (LD); (**d**) combined pendant/sessile drops. Containerless measurements using the oscil lating drop method in electromagnetic levitation OD-EMLµ g: (**e**) parabolic flight (PF) and (**f**) on board of the International Space Station (ISS) together with (**g**) Temperature–Time Diagram of an os cillating drop cycle. Metal drop (**h**) in (LD) measurements and (**i**) SEM micrograph of RENE N5® solidified alloy drop after surface tension measurements. Surface tension isotherms (**j**) of liquid Ni Al alloys for T=1923 K calculated by 1—Compound formation model; 2—Quasi chemical approxi mation for a regular solution and 3—Ideal solution model. An enlarged area (**k**) of Ni-Al surface tension isotherms (**j**) with the respect to Al-content in Ni-based superalloys is shown [210,218,219,220,223,224].

Among the thermophysical properties of liquid Ni-based superalloys, we also measured the surface tension, using the sessile (SD) and large drop (LD) container-based experimental methods [218,219,220] (Figure 30). However, none of container/substrate materials are inert when in contact with metallic melts having a high melting temperature, and thus, to reduce the formation of reaction products at the metal/container interface, preliminary wetting experiments have been performed. In this way, the selection of an appropriate inert crucible/substrate material as well as the choice of suitable environmental atmosphere are preeminent issues to obtain reliable property data. In addition, in the case of Ni-based alloys, alternatives for container-based methods, i.e., containerless processing techniques [221,222,223,224], were used (Figure 30). The measurements were performed under microgravity conditions using the oscillating drop (OD) method in an electromagnetic levitation: on ground (OD-EML^g^), on board a parabolic flight (PF) airplane [219], and on board (OD-EML^μ-g^) the International Space Station (ISS).

All the experimental surface tension data were used for the validation of the theoretical models. In order to identify the potential sources of systematic error and to arrive at the best-agreed-upon values, round robin measurements of the same property in different laboratories and with different methods or equipment have been performed. Results on the surface tension measurements and modeling of liquid Ni-based superalloys alloys are shown in Figure 30. An example of thermophysical properties datasheet with recent experimental data of the CMSX-10 liquid alloy, often used in databases, is shown in Table 5.

## 7. Amphiphobic Coatings for Environmental Protection of Solar Panels

**Figure 31:**
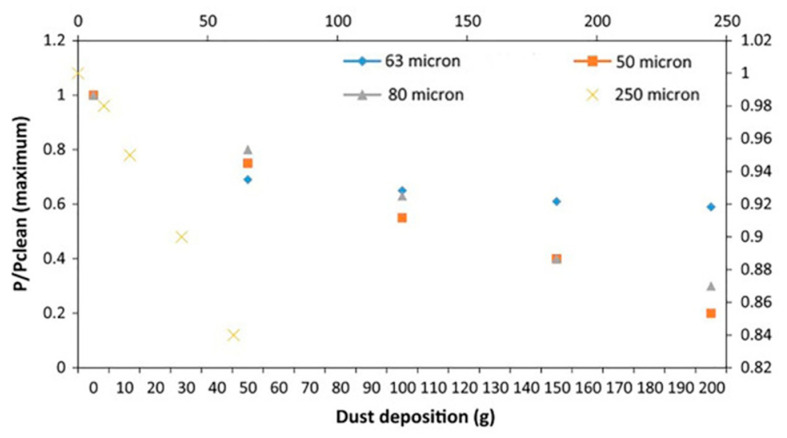
PV power output as function of dust deposition. Reprinted with permission from ref. [229].

Among the various practical applications of renewable energy, photovoltaic (PV) systems have become the main way to utilize solar energy due to their high efficiency, safety, and environmental friendliness [225]. With the diversification of PV module installation areas, PV modules will not only have to face exposure to high humidity and UV radiation but also the problem of dust and sand deposition [226]. A key factor contributing to the decreased efficiency of solar panels is the accumulation of soiling substances, such as dust, which leads to higher maintenance costs and accelerated material degradation, particularly in solar power plants [227]. Since extra urban sites (deserts or wastelands) are more frequently chosen as a location for large solar plants, avoiding dust accumulation problems can produce up to 30% power decrease, as in Figure 31, and at the same time, human intervention cannot be a solution [228].

Therefore, there is an urgent need to maintain the conversion efficiency of the PV through effective techniques such as reducing reflectivity and dust deposition. To cope with such a scenario, implementing surfaces with highly water-repellent coatings, with superhydrophobic and amphiphobic properties, offers a potential solution, as their self-cleaning ability helps minimize the accumulation of dirt and debris.

For solar panels, amphiphobic and self-cleaning coatings must be both transparent and long-lasting to reduce the need for human intervention and maintenance while ensuring high efficiency. In recent years, nanostructured superhydrophobic (SHS) coatings (water contact angle >150°) with high optical transparency (85–95%) have been developed to enhance self-cleaning and optical performance of photovoltaic (PV) modules.

**Figure 32:**
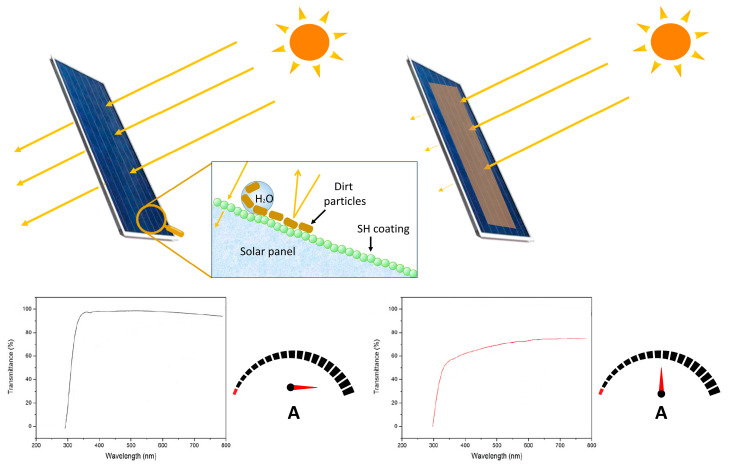
Comparison between high-transmittance superhydrophobic-coated (**left**) and standard (**right**) solar panels in terms of module efficiency.

Here, we provide key elements of the application of amphiphobic, high-transmittance, self-cleaning coating solutions in Figure 32 [230].

In order to obtain a superhydrophobic surface, we followed different routes, namely working on surface energy by exploring novel materials and on its surface morphology and roughness. As transparency is linked to light scattering, which increases with surface roughness, surface roughness is generally kept below 100 nm to minimize scattering. However, working with modulating material surface roughness does not always result in highly transparent superhydrophobic and amphiphobic coatings [231].

Exploring materials to tailor surface energy usually involves toxic fluorine-containing materials with low surface energy, which inevitably raises health and environ mental concerns and increases production costs. As an example, a coating was developed using the aerosol-assisted chemical vapor deposition of polytetrafluoroethylene (PTFE), achieving a CA of 168.3° with excellent optical transparency (92% at 350 nm, stable across the visible range), and remarkable durability against sand impact and water droplet erosion [232].

**Figure 33:**
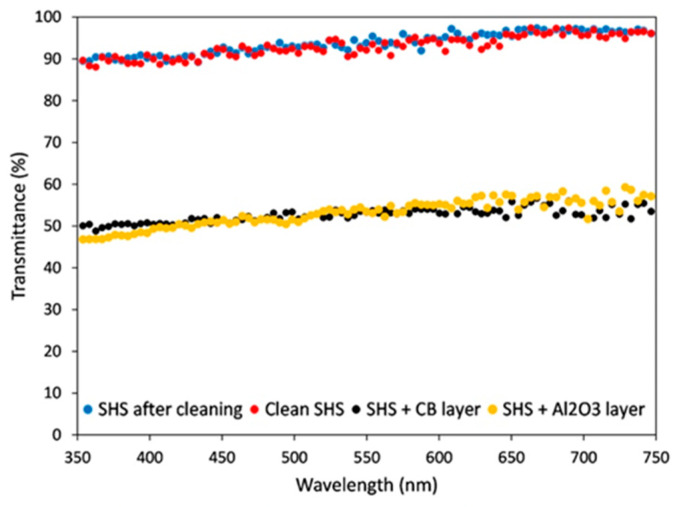
Transmittance spectra of clean SH coating (red dots), SH coating dirtied with CB and Al2O3 (black and yellow dots), and SH coating after cleaning (blue dots).

Jeong et al. [233] employed PECVD to fabricate a silica nanoparticle array (SNA) layer on a PET substrate, functionalized with fluorinated silane (PFOTS), reaching a water CA of 169° and transmittance between 65 and 80%, while also boosting organic solar cell efficiency by 13%. Studies are carried out to prepare stable, robust, transparent, and nonfluorinated superhydrophobic coating films. As an example, combustion chemical vapor deposition is used to grow secondary nanostructures on etched metal nanopillars, creating a superomniphobic surface with water, oleic acid, and hexadecane CAs of 172°, 163°, and 153°, respectively, and a transmittance ranging from 91.5% to 94.5% in the 400–700 nm range [234]. A recent study [235] presented a transparent, fluorine-free superhydrophobic coating with CA ~157°, strong optical clarity, long-term durability, and self-healing properties recovering mechanical damage when heated to 80 °C. A field trial conducted in Egypt in ref. [236] evaluated a self-cleaning nano-coating deposited onto PV panels against dust accumulation under harsh desert climate. This coating shows that, over a 10-month outdoor exposure, the coated panel exhibited a 64.7% increase in the short-circuit current and a 65.2% rise in the maximum power output compared to an uncoated reference, maintaining a higher operational efficiency throughout the day (12–13.5%) versus the uncoated one (7–8%). We also developed [237] a transparent hybrid organic–inorganic superhydrophobic coating using a spray technique, based on silica and Al_2_O_3_ nanoparticles with a polymeric shell (CA > 170°), high transparency (up to 94% at 350 nm), and strong self-cleaning ability. The coating was tested under various simulated environmental conditions, including acid rain and harsh environment (pH 1–13) and water droplet impact (over 50,000 impacts without losing performance), showing also the ability to fully recover after durability and soiling tests (Figure 33).

Although anti-smudge coatings for photovoltaic glass have advanced, their mechanical durability and corrosion resistance remain inadequate. Therefore, in the field of anti-reflective coatings and anti-smudge surfaces, it is crucial to concentrate on several key research areas, including engineering multilayer coatings, coatings that integrate multiple functions (e.g., anti-reflection, anti-smudge, anti-icing, and self-healing), and designing gradient refractive index structures and introducing crosslinking agents.

Given the worldwide spread of large solar plants, the potential commercial upscale of such self-cleaning and transparent coatings is highly demanded. Their comparatively low cost and easy-to-maintain features make them good candidates, provided that the present limitations, like durability data, can be overcome in the long term.

## 8. Life Cycle Assessment Applied to the AlTiN Case Study

Life cycle assessment (LCA) is a comprehensive quantitative method designed to evaluate the potential environmental impacts of energy materials throughout a product’s entire life cycle. A “cradle-to-grave” approach considers all production phases, from the extraction of raw materials to production, transport, and distribution, all the way to its use phase and eventual end of life, which includes waste treatment and potential recycling. A distinct, yet common, approach, often applied in scientific research, is the “cradle-to-gate” analysis. This approach specifically aims to pinpoint environmentally critical steps during the development of new production or synthesis routes, omitting the use and end-of-life phases from its scope.

Literature studies demonstrated the importance of an early environmental evaluation for a new material development, also at the laboratory scale, permitting to minimize the final impact in the case of a pilot and industrial upscaling [238,239,240]. LCA applied to laboratory material development is conducted as a preliminary measure to identify environmental hotspots within innovative processes and emerging applications at low technology readiness levels [241,242,243,244,245]. LCA studies can also prove instrumental in pinpointing environmentally critical stages and supporting the development of optimized experimental setups, thereby contributing to more sustainable laboratory operations. [246]

Here, we show the example of LCA applied to an experimental campaign involving the materials presented in Section 4, namely AlTiN-based coatings on commercial steel sheets produced by reactive DCMS as HPB films for mitigating hydrogen embrittlement [247]. As reported in Section 4, this peculiar nitride is considered a suitable material for different applications, including hydrogen permeation barriers, and its PVD magnetron sputtering method of synthesis is a consolidated synthesis technique for thin-film deposition, which allows performing an affordable LCA even at low technology readiness level. This approach can provide valuable insights leading to the development of more sustainable deposition strategies to proceed towards environmentally responsible material innovation. The aim of this LCA study was to integrate sustainability into the laboratory-scale DCMS deposition of AlTiN-based thin films, offering valuable insights to support the design of subsequent research campaigns or instrumental setups with reduced environmental footprints. Primary data from the DCMS deposition process, including electricity consumption, quantity and type of solvents, gas and other inputs, and quantity and type of wastes and emissions, were collected directly from experiments conducted at the National Research Council (CNR) laboratories in Padua (Italy) between 2017 and 2018 [248]. Secondary data were retrieved from the Ecoinvent 3.5 database, with a good temporal representativeness (data from 2004 to 2018). The Functional Unit (F.U.) was 50 coated samples, each one with an area of 25 cm^2^ and an AlTiN thickness of 3 µm. The DCMS process used to prepare 3 µm AlTiN coatings was modeled through eight distinct process units (“Target”, “Substrate”, “Cleaning”, “Gases”, “Power Supply”, “Heating”, “Vacuum”, and “Cooling”).

**Figure 34:**
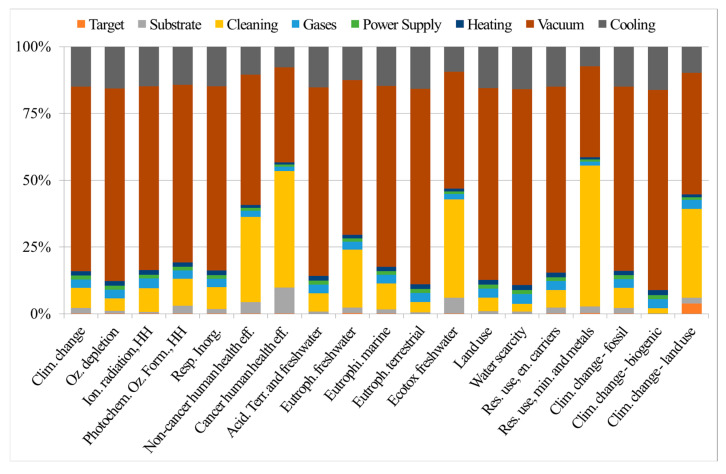
Contribution analysis of characterized results calculated with EF 2.0 method. F.U.: 50 AlTiN-coated samples (1 sample at specific time).

The life cycle impact assessment method applied was environmental footprint (EF) 2.0. The complete inventory analysis of this work can be found in ref. [249]. Figure 34 shows the contribution analysis of characterized results referred to as the F.U. of the study, assuming to coat a substrate at a specific time.

**Figure 35:**
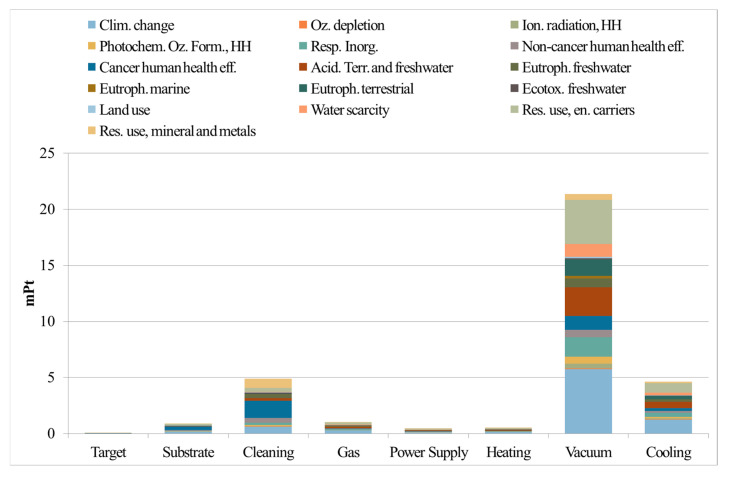
Single score of characterized results after normalization and weighting with EF 2.0 method. F.U.: 50 AlTiN-coated samples (1 sample at specific time).

The most impacting activity was in the “Vacuum” system, followed by the “Cooling” and “Cleaning” phases; their relevance was confirmed by the single score values (characterized results after normalization and weighting) shown in Figure 35, contributing to the total impacts with 63%, 14%, and 14%, respectively. “Climate Change”, “Resource Use”, “Acidification” (“Terrestrial” and “Freshwater”), and “Cancer Human Health Effects” were the most relevant impact categories with a contribution of 25%, 17%, 11%, and 10%, respectively.

As similarly reported in previous studies [250,251,252], the results confirmed that the main driver of environmental impacts in the investigated laboratory activities is the use of electricity for the operation of instruments (83%), such as reaching a low pressure. Conversely, the impact from consumable materials is negligible. Being aware of these hotspots, several optimization strategies may be adopted to reduce the overall electricity consumption and, thus, the respective environmental impacts. These include using a prechamber for sample loading or designing a deposition system that can permit to simultaneously coat a higher number of samples.

Thus, several materials have a high potential to be the resources of the energy of the future. However, despite these great prognostics for sustainable materials, LCA demonstrated that they are not necessarily environmental-impact-neutral, and their potential to be less-energy consuming or less-polluting than other materials must be assessed considering each specific case by means of tools like LCA.

## 9. Recycle and Reuse of Materials for Energy

The concept of circularity must be applied to all materials for energy, as the deployment of solar panels, energy storage and conversion systems, and wind turbines increases and the end-of-life management of these components becomes increasingly pressing.

The presented materials for energy comprise a wide range of materials that intersect with the topic of life cycle of materials. Most of the described solutions are in an early stage of development; nonetheless, the topic of how these materials will be implemented in the energy value chain and to which extent they will affect the overall carbon footprint of the specific application are already taken into consideration, by applying the LCA (as performed in the previous section) at the laboratory scale. The material recovery and reclamation by mechanical and chemical processes are simpler when the material is used as such in the energy devices (e.g., silicon in solar panels), as they can be reclaimed and reintroduced in the same supply chain. Refurbishment and remanufacturing are criteria applied to wind turbines, where wind turbine blades can be repaired and refitted. As wind energy grows, finding a way to chemically deconstruct and reuse the composites in wind turbine blades and effectively recycle structural materials is a serious concern and very energy-intensive; hence, more efforts to study the recycling and reuse of the wind turbine blades are essential. In these frameworks, metals, which we have been described in Section 3, Section 4 and Section 6, represent a special category; the high impact of the production of pure metals from the mineral sources is a well-known problem. At the industrial level, metal reuse and recycle is a topic already being developed for many metals and alloys. Indeed, the reuse of metals from the recycling process is still in evolution; the performances of recycled metals have to be verified due to the different amounts and types of impurities in ore metals or recycled metals, which can be obstacles to their use in specialty applications. A combination of metals with other materials can indeed decrease the extent of recyclability of the former and need consideration not only at the scientific level but also at the social and economic levels (sorted waste collection, regulatory restrictions, and carbon footprint of the recycling process, just to name few of them).

**Figure 36:**
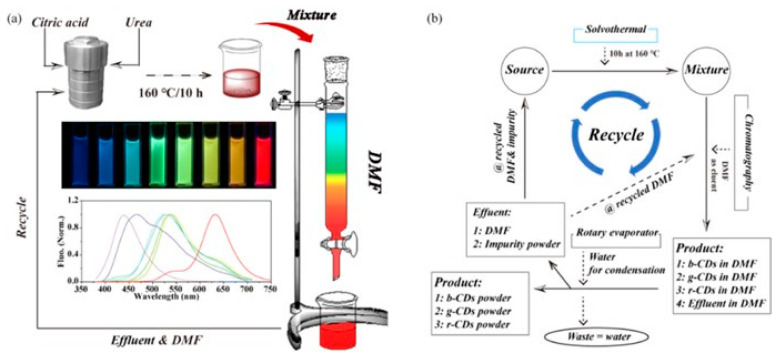
(**a**) Schematic illustration of recycling preparation process of multicolor fluorescent CDs. (**b**) Diagram for recycling preparation process. Reprinted with permission from ref. [253]. Copyright 2022 American Chemical Society.

As for the materials reported in Section 2, it remains challenging to develop approaches for the recycling and reuse of CDs, and the literature on this subject is scarce. There are two main reasons for this. The first, more obvious, reason is that CDs are considered valuable products obtained from waste. This fosters the valorization of waste materials through reuse and mitigates their potential adverse environmental impacts. For example, Fan et al. [23] recycled graphite from spent lithium-ion batteries (LIBs) to create graphite-decorated CDs (CDs@Gra), which are then reused as electrode materials in energy storage devices such as lithium/sodium-ion batteries and supercapacitors. The second reason is less evident and relates to the fact that CD production is mainly limited to the laboratory scale. In some cases, these approaches are characterized by low reaction yields and large amounts of unreacted precursors and reaction intermediates. This limits their application to large-scale industrial production due to concerns about cost and pollution. Developing strategies to reuse these products could improve CD reaction yields and make their production more sustainable. In this regard, Shan et al. [253] reported a method for producing CDs in which the unreacted precursors (effluent) and solvent, reclaimed from the evaporated CD solution, are reused as the reaction source and eluent, respectively, in the next preparation cycle (see Figure 36). This process aims to provide an environmentally friendly route for future industrial mass production and advance CD applications.

In light of all the above considerations and the significant interest shown by the scientific community in adopting the principle of circular economy when developing materials for energy, recycling and reuse will be a challenging aspect of future research studies on these materials. Indeed, it must also be said that the scientific challenges pertaining to the burden of cost play a crucial part in the advancement of recycling materials used in renewable energy and energy storage systems.

## 10. Conclusions and Perspectives

The escalating global energy demand and the imperative for decarbonization necessitate innovative solutions to enhance energy efficiency, manage carbon emissions, reduce the costs of renewable energy, and develop scalable technologies for integrating renewables into the energy distribution system. Global cooperation and policy support are essential for addressing environmental challenges and ensuring long-term sustainability. Here, we have provided some examples of new energy storage and conversion materials that present several key benefits, offering promising methods to tackle challenges related to energy generation, on-demand supply, pollution remediation, and advanced manufacturing with minimized costs and environmental footprints. Despite significant advancements in PCM research and applications, further research is required to advance thermal energy storage technologies, which are crucial for achieving climate neutrality goals and ensuring a more flexible, efficient, and sustainable energy system. The adoption of ductile iron castings in the wind energy industry represents a step towards making renewable energy more accessible, reliable, and resilient. Continued innovation and collaboration could position ductile iron as a cornerstone material in the pursuit of a sustainable planet. While ferroelectric nanocomposites for energy storage show promise, challenges such as ensuring consistent nanoscale morphology, optimizing composite compositions, and addressing interface compatibility issues remain. Additionally, developing scalable and cost-effective fabrication methods and processes is necessary for their commercial viability. To rationally select a technology, the capital and operation costs and life cycle assessment of the whole technical system must be considered.

As for the commercial exploitation of the proposed materials, the following considerations provide a perspective on their market:

- The synthetic approaches of C-based nanostructures are still limited to the laboratory scale; consequently, the commercialization of CDs is limited to few companies that offer small amounts of customized CDs for laboratory applications in their catalogues.
- Applications of PCMs are traditionally dominated by the use of organic compounds and inorganic salts. However, the recent commercial developments and industrial applications of metallic PCMs are focused on specific sectors, where their unique thermal properties could provide significant advantages. As an example, they can be employed in the energy storage at high temperature in concentrated solar power plants or for the recovery of waste industrial heat generated by metallurgical or cement plants. Moreover, metallic PCMs can be integrated into power-to-heat technologies that store the excess electricity by transforming it into thermal energy.
- As for ferroelectric materials, although they offer valuable properties for both commercial and domestic applications, their target market faces significant challenges, primarily due to the high costs of raw materials and complex production processes that hinder widespread adoption. Additionally, strict environmental regulations, especially concerning lead-based compounds, are driving the shift toward more environmentally sustainable alternatives, influencing market dynamics. To address current challenges, ongoing research is also increasingly focused on cost-effective, eco-friendly solutions, such as ferroelectric composites, for next-generation applications in flexible electronics, sensors, and energy-efficient systems, promoting a more scalable and sustainable evolution within this emerging market segment.
- Geopolitical instability, fluctuations in raw material prices, and logistics bottlenecks can affect the timely delivery and cost of cast iron components. The trend toward ever-larger wind turbines drives demand for correspondingly larger castings, pushing the technical limits of foundry capabilities.
- As for commercialization pathways of the metallic-based systems for hydrogen barriers and membranes, the key factors to be considered are that barrier layers produced by PVD or CVD require that these processes are scaled for larger components and more complex geometries; cost considerations could also be perceived as a barrier to commercial exploitation; however, while initial costs may be higher than for traditional coatings, the durability and performance benefits are proving attractive in demanding environments. Furthermore, standardization and certification have to be addressed to certify intermetallic-based materials for use in critical infrastructure, ensuring reliability and interoperability.
- Given the worldwide spread of large solar plants, the potential commercial upscale such self-cleaning of transparent coatings is highly demanding. Their comparatively low cost and easy-to-maintain features make them good candidates, provided that the present limitations, like durability data, can be overcome in the long term.

In conclusion, the selection of energy technologies must consider capital and operational costs, commercialization potential, and the life cycle assessment of the entire technical system. Looking ahead, several emerging trends and research directions are evident from these contributions. The integration of artificial intelligence with material design, the development of multifunctional materials for energy applications, and the optimization of sustainable production methods represent particularly promising avenues for future research. Furthermore, the principles of a circular economy—repair, reuse, and recycling of materials—should be integrated into energy technology development to enhance sustainability. Despite the remaining challenges, researchers are diligently working to overcome obstacles by developing better materials. Effective energy management significantly impacts global warming and human health, necessitating continuous attention from policymakers to sustain strategies for achieving both economic growth and climate protection coordinated internationally. Clearly, this is not a journey that scientists should undertake alone. Effective energy management has a significant impact on global warming and human health, necessitating continuous attention from policymakers to sustain strategies for achieving both economic growth and climate protection.

## Figures and Tables

**Table 1:** Comparison of different PCM classes. Reprinted with permission of ref. [58] (Copyright 2024 Elsevier Ltd.).

Type of PCM	Paraffin	Non-Paraffin	Sugar Alcohols	Hydrated Salts	Molten Salts	Metal-Based
Latent heat (J g^−1^)	140–260	45–200	0–350	100–300	60–1044	16–1800
Thermal conductivity (W m^−1^ K^−1^)	0.22–0.82	0.1–0.2	0.37–1.4	0.15–1.0	<1.0	1–430
Melting point (°C)	−10–70	−5–70	0–250	8–90	200–1000	−40–1400
Advantages	- Low cost - Abundant - Good thermal stability - Non-toxic - No super-cooling	- Renewable source - Less flammable - Less supercooling	- Can form solid/solid PCM - Low cost	- Non-flammable - Low cost - Non-toxic	- High thermal stability - High operation temperature - High latent heat	- High thermal conductivity - Wide latent heat range - Good thermal stability
Disadvantages	- Flammable - Volume change during thermal cycle	- Less chemically stable than paraffin - Expensive - Unpleasant odor	- Poor thermal stability - Supercooling - Can undergo polymorphic changes	- Supercooling - Corrosion - Loss of water upon cycling - Incongruent melting	- Corrosion - Hygroscopic	- Heavy - Expensive - Corrosion

**Table 2:** Reviewed publications of metallic PCM production: type of metal, temperature of phase change, and main process features.

Approach	Strategy	Processing State *	Materials PCM/Container	PC Temperature	Ref
Single container	Induction melting	L	Al34%Mg6%Zn alloys/SS, CS	450 °C	[69]
	Furnace melting	L	Mg_84_Cu_16_ and Mg_59_Cu_41_ (% at.) eutectic alloys/SS	488 °C 550 °C	[70]
	Furnace melting	L	Cu–Ge alloy/steels and ceramics	644 °C	[71]
	Furnace melting	L	Al-(0–25% wt) Si/Al_2_O_3_	577 °C	[68,72]
	Furnace melting	L	Mg–Cu alloys/SS	485 °C	[73]
	Melting	L	Pb/SS Al-(0, 12.6, 25.1) %Si/SS	230 °C 660–570 °C	[74]
	N.A	N.A.	Al–Si alloy/ceramic container	585 °C	[75]
	Sim	L	Al, Pb, Sn/SS	660 °C Al, 327 °C Pb, 231 °C Sn	[76]
Encapsulation	ALD	S	encapsulated Sn nanoparticles/oxide layers (SiO_2_, Al_2_O_3_)	232 °C	[77]
	CVD	S	Fe particles/SiC/C-shells	1136 °C	[78]
	Micro-encapsulation	M	Al-Si alloy particles/Al_2_O_3_	573 °C	[79]
	Electroplating method	S	Cu ball/other metals	1083 °C	[64,80]
	ADL	L	Fe–Cu alloys/Fe oxide	1083 °C	[81]
Self-encapsulation	Self-encapsulation (Sim)	M	Al–Si shot/Si	577 °C	[82]
Containerless	Simple powder metallurgy	S	Al-Sn, Fe-Cu	232 °C Al-Sn, 1085 °C Cu-Fe	[83]
	Casting-water granulation	L	Al, A356, Al-(40% wt) Sn, A356-(40% wt) Sn alloys	231 °C	[84]
	Powder bed laser melting	L	Al-40% wt Sn Miscibility Gap Alloy	231 °C	[85]
	Powder metallurgy and ball milling	S	Al−Sn alloy	231 °C	[86]
	Powder metallurgy	S	Al-Sn system	230 °C	[87]
	Casting	L	Al_70_Bi_10_Sn_20_ alloy	200 °C	[88]

* Legend: ALD: atomic layer deposition; CVD: chemical vapor deposition; ADL: aerodynamic levitation;; L: liquid state; S: solid state; M: mixed solid-state steps and liquid-state steps; SS = stainless steel; CS = plain steel or carbon steel.

**Table 3:** Ductile iron wind mill castings.

Assembly	DI Parts	Average Weight (ton/MW)
Rotor system	Hub, blade, adapter, bearings	6.44 (hub: 4.5 ton/MW)
Shaft	Shaft, bearings	0.44
Turbine Frame	Nacelle, bed plate, yaw ring	3.85 (nacelle: up to 10 ton)
Gear Box	Housing, support, bearings	1.71
Others		1.00
TOTAL		13.44

**Table 4:** EN-GJS-400-18 LT specification for wind mill castings.

Property	Metric	Imperial
Tensile Strength	400 MPa	58 ksi
Yield Strength	240 MPa	35 ksi
Elongation	18%	18%
Hardness	160−170 BHN	160−170 BHN
Impact Energy at 20 °C (−4 °F) Average of 3 tests	12 J	8.8 ft∙lb
Minimum (1 specimen)	9 J	6.6 ft∙lb

**Table 5:** An example of datasheet describing nominal composition (in wt. %) and the parameters related to the thermophysical properties data (Specific heat capacity; Density; Surface tension; Viscosity) of the CMSX-10 liquid alloy. The abbreviations indicate the measurement methods used: Modulation Calorimetry in Electromagnetic levitation (MC-${EML}^{\mu -g}$); Differential Scanning Calorimetry (DSC); Oscillating Drop method in Electromagnetic levitation (OD-${EML}^{\mu -g}$); Pinned Drop (PD) method; Modified Sessile Drop Method (MSDM); Oscillating drop method in electromagnetic levitation (OD−EML) on parabolic flights (*PF*). Superscripts: *μ* − *g*—microgravity.

Alloy Ref.	Ni	Cr	Co		Mo	W	Nb	Ti	Al	Ta	Re	Hf	Other Elements
CMSX-10 [224]	69.57	2.0	3.0		0.4	5.0	0.1	0.2	5.7	8.0	6.0	0.03	−
CMSX-10 [220]	69.70	2.0	3.0		0.4	5.0	0.1	0.2	5.7	8.0	6.0	0.03	Bal = −0.03
	**Liquidus Temp.** $T_{L}$ **,** K				**Specific Heat Capacity** $C_{p}$**,** $J({gK)}^{-1}$			**Measurement Temperature** **Range, K**		**Method**			**Exp. Error** **%**
CMSX-10 [224]	1706 ± 5				0.71 ± 0.05			1650−1775		MC-${EML}^{\mu -g}$			1.0
CMSX-10 [220]	1702 ± 4				0.7			1690−1730		DSC			±10.0
	**Liquidus temp.** $T_{L}$ **,** K		**Density** $\rho_{0}$ **at** $T_{L}$**,** g**·**${cm}^{-3}$		**Temperature coefficient** dρ/dT**,** $10^{-3}g$ **·** $cm^{-1}K^{-1}$			**Measurement** **temperature** **range,** K		**Method**			**Exp. Error** **%**
CMSX-10 [224]	1706 ± 5		8.21 ± 0.15		−0.535 ± 0.05			1525−1875		OD-${EML}^{\mu -g}$			1.6
CMSX-10 [220]	1702		7.19 ± 0.03		−1.6			1715−1773		PD			±5.0
CMSX-10 [209]	1676		8.08		−0.661			1676−1825		MSDM			±0.88
	**Liquidus temp.** $T_{L}$ **,** K		**Surface** **tension** $\sigma_{0}$ **at** $T_{L}$ **,** $Nm^{-1}$		**Temperature coefficient** dσ/dT, $10^{-4}Nm^{-1}K^{-1}$			**Measurement temperature range,** K		**Method**			**Exp. error** **%**
CMSX-10 [220]	1702		1.758 ± 0.025		−4.7 ± 0.4			1715−1773		PD			<3
CMSX-10 [223]	1683		1.71 ± 0.02		−5.80			1575−1925		PF:OD−EML			<1
CMSX-10 [224]	1706 ± 5		1.698 ± 0.002		−1.438 ± 0.294			1575−1825		$\mathrm{OD-}{EML}^{\mu -g}$			<±1
	**Liquidus temp.** $T_{L}$**,** K		**Viscosity** $\eta (T_{L})$ **,** mPa·s	**Viscosity** $\eta_{0}$ **,** mPa·s	**Activation** **energy** $∆E_{A}$ **,** eV/atom			**Measurement temperature range,** K		**Method**			**Exp. error** **%**
CMSX-10 [223]	1683		7.7 ± 1.2	0.23	0.16			1600−1840		PF:OD−EML			±15
CMSX-10 [224]	1706 ± 5		8.6	0.406	0.449			1600−1780		$\mathrm{OD-}{EML}^{\mu -g}$			1.9

## Abbreviations

The following abbreviations are used in this manuscript:

ADI	Austempered Ductile Iron
AM	Additive manufacturing
BDS	Breakdown strength
CA	Contact angle
CALPHAD	Calculation of Phase Diagrams
CDs	Carbon-based dots
CVD	Chemical vapor deposition
CHG	Chunky graphite
DCMS	Direct-Current Magnetron Sputtering
DI	Ductile iron
DSC	Differential scanning calorimetry
EDX	Energy-Dispersive X-ray Spectroscopy
FE	Ferroelectric
FTO	Fluorine-doped tin oxide
GHG	Greenhouse gas
HE	Hydrogen embrittlement
HER	Hydrogen evolution reaction
HiPIMS	High-power impulse magnetron sputtering
HPB	Hydrogen permeation barriers
LCA	Life cycle assessment
LHS	Latent heat storage
LPBF	Laser Powder Bed Fusion
LSVs	Linear sweep voltammetries
MWCNT	Multi-wall carbon nanotube
OER	Oxygen evolution reaction
PCM	Phase-change material
PE	Photothermal evaporation
PE-CVD	Plasma-enhanced chemical vapor deposition
PHE	Photocatalytic hydrogen evolution
PV	Photovoltaic
RFE	Relaxor ferroelectric
SEM	Scanning electron microscopy
SIMS	Secondary ion mass spectrometry
TEM	Transmission electron microscopy
TES	Thermal energy storage
XRD	X-ray diffraction
